# Naphthoquinones as Covalent Reversible Inhibitors of Cysteine Proteases—Studies on Inhibition Mechanism and Kinetics

**DOI:** 10.3390/molecules25092064

**Published:** 2020-04-28

**Authors:** Philipp Klein, Fabian Barthels, Patrick Johe, Annika Wagner, Stefan Tenzer, Ute Distler, Thien Anh Le, Paul Schmid, Volker Engel, Bernd Engels, Ute A. Hellmich, Till Opatz, Tanja Schirmeister

**Affiliations:** 1 Department of Chemistry, Organic Chemistry Section, Johannes Gutenberg-Universität, Duesbergweg 10-14, 55128 Mainz, Germany; klein@uni-mainz.de; 2 Institute of Pharmaceutical and Biomedical Sciences, Johannes Gutenberg-Universität, Staudingerweg 5, 55128 Mainz, Germany; barthels@uni-mainz.de (F.B.); pajohe@uni-mainz.de (P.J.); 3 Department of Chemistry, Biochemistry Section, Johannes Gutenberg-Universität, Johann-Joachim Becherweg 30, 55128 Mainz, Germany; a.wagner@uni-mainz.de (A.W.); u.hellmich@uni-mainz.de (U.A.H.); 4 Institute of Immunology, University Medical Center, Johannes Gutenberg-Universität Mainz, Langenbeckstr. 1, 55131 Mainz, Germany; tenzer@uni-mainz.de (S.T.); ute.distler@uni-mainz.de (U.D.); 5 Institute of Physical and Theoretical Chemistry, Universität Würzburg, Emil-Fischer-Straße 42, 97074 Würzburg, Germany; thien_anh.le@physik.uni-wuerzburg.de (T.A.L.); paul.schmid@uni-wuerzburg.de (P.S.); volker.engel@uni-wuerzburg.de (V.E.); bernd.engels@uni-wuerzburg.de (B.E.); 6 Centre for Biomolecular Magnetic Resonance (BMRZ), Goethe-University Frankfurt, 60323 Frankfurt, Germany

**Keywords:** protease, rhodesain, covalent reversible inhibition, 1,4-naphthoquinone, nucleophilic addition, prodrug

## Abstract

The facile synthesis and detailed investigation of a class of highly potent protease inhibitors based on 1,4-naphthoquinones with a dipeptidic recognition motif (HN-l-Phe-l-Leu-OR) in the 2-position and an electron-withdrawing group (EWG) in the 3-position is presented. One of the compound representatives, namely the acid with EWG = CN and with R = H proved to be a highly potent rhodesain inhibitor with nanomolar affinity. The respective benzyl ester (R = Bn) was found to be hydrolyzed by the target enzyme itself yielding the free acid. Detailed kinetic and mass spectrometry studies revealed a reversible covalent binding mode. Theoretical calculations with different density functionals (DFT) as well as wavefunction-based approaches were performed to elucidate the mode of action.

## 1. Introduction

Covalently acting drugs have traditionally been considered as problematic due to their potential to elicit off-target effects. In the last years, however, such compounds have received increasing attention in drug development due to several advantages including longer target residence times, higher potency and ligand efficiency. It has been recognized that the benefits of such inhibitors can be additionally enhanced by careful tuning of their reactivity, i.e., by switching the covalent inhibition mode from covalent irreversible to covalent reversible [1,2,3,4,5,6,7,8].

In general, covalent inhibitors consist of two parts, namely a recognition unit fitting into the substrate binding pockets, and an electrophilic functional group. In the case of protease inhibitors, the recognition unit is typically a peptidic or peptidomimetic fragment. Covalent inhibitors also contain an electrophilic functional group (the so-called “warhead”), which can undergo a chemical reaction with a nucleophilic amino acid in the vicinity. In most cases, Cys, Ser or Thr side chains are addressed. A large fraction of the known types of warheads targeting Cys residues is based on activated double bonds (Michael acceptors) [9] comprising, e.g., vinyl sulfones [10,11,12], α,β-unsaturated esters, nitriles and ketones [13,14,15,16]. Depending on the substitution pattern of the double bond, the inhibition of the target enzyme can either be covalent reversible or irreversible [17]. Heterocyclic or peptide-derived nitriles constitute another important substance class of protease inhibitors, which inhibit cysteine and serine proteases in a covalent reversible manner via the formation of a (thio) imidate [18,19,20,21].

In the present study, we compared chloro- and nitrile-substituted peptidyl 1,4-naphthoquinones with respect to their inhibition properties. In principle, such multifunctional warheads [22] could undergo either Michael-type additions or substitution reactions or—in case of the nitrile derivative—also reversible addition to the nitrile group.

We explored the potential of such groups as new warheads for cysteine proteases of the papain family, namely the human cathepsins L and B (Cath. L and Cath. B), and the cathepsin L-like *Trypanosoma* protease rhodesain (Rhod.). The human enzymes of this family play crucial roles in, e.g., tumor metastasis [23,24]. Rhodesain from *Trypanosoma brucei rhodesiense*, the parasite causing human African trypanosomiasis (HAT), is essential for the parasite’s survival as it is involved in several host invasion processes [25,26]. In order to check the selectivity, the inhibition potencies of the new inhibitors were also tested for the serine protease of the Dengue virus (DENV PR) [27,28].

Since we were interested in the inhibition properties of the two warheads, we used the same recognition unit, the dipeptide sequence H_2_N-l-Phe-l-Leu-OBn and attached the 1,4-naphthoquinone unit to its N-terminus. This dipeptide sequence was chosen since it was previously shown to be an appropriate recognition unit for cathepsin-like cysteine proteases [29,30]. Moreover, as opposed to amino acids with functionalized side chains, no additional protection/deprotection steps are necessary during synthesis. We previously observed that rhodesain is able to hydrolyze compounds with the dipeptidic H_2_N-l-Phe-l-Leu-OBn recognition unit [30]. In agreement with these findings, we also observed the protease-mediated hydrolysis of the benzyl ester moieties of the naphthoquinone-modified inhibitors to yield the free acids (see Section 2.4 and Section 2.6). We therefore additionally compared the free acids as well as the respective methyl and *tert*-butyl esters of our current molecules for their ability to inhibit rhodesain.

Mass spectrometry revealed the formation of a covalent bond between the inhibitors and rhodesain. We therefore also computed the thermodynamics of possible inhibition reactions to gain insights into the energetics of the various possible covalent inhibition mechanisms. The chemical properties of such groups are strongly influenced by their degree of aromatic stabilization, which is often overestimated by standard density functional approaches [31]. To evaluate which theoretical approaches are sufficiently reliable to predict the inhibition mechanisms and potencies of such groups, a benchmark study employing various wave functions as well as density-based approaches were performed.

## 2. Results

### 2.1. Syntheses

**Scheme 1:**
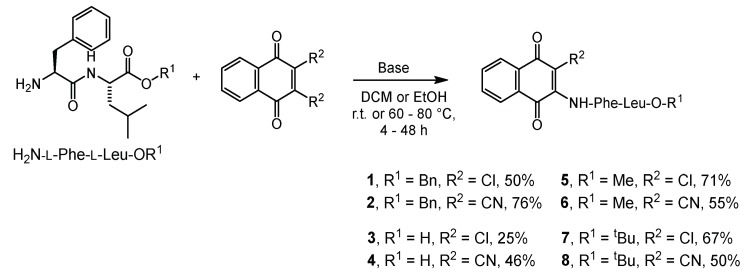
Syntheses of potential protease inhibitors with naphthoquinone units as the reactive group.

The dipeptide product H_2_N-l-Phe-l-Leu-OBn was synthesized as reported previously [30]. The potential inhibitors were synthesized by an addition/elimination reaction on the naphthoquinone unit carrying two potential leaving groups (Scheme 1).

The respective methyl and *tert*-butyl esters **5**–**8** were synthesized accordingly. To further investigate whether the hydrolysis products of the benzyl esters **1** and **2** are the active compounds as indicated by the MS and hydrolysis studies (see Section 2.4 and Section 2.6), the corresponding free acids **3** and **4** were also synthesized. 

### 2.2. Enzyme Assays

**Figure 1:**
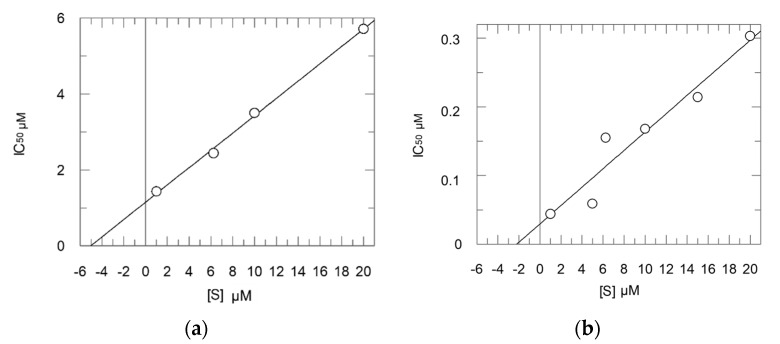
Dependence of inhibition potency (IC_50_ values) from substrate concentration for inhibition of cathepsin L by compounds **a** (left) and **b** (right). Increasing IC_50_ values at increasing substrate concentrations prove competitive inhibition; the *K*_i_^(*)^ value (corresponding to the IC_50_ value at zero substrate concentration) is obtained as the intercept of the regression line with the *y*-axis (**a**: *K*_i_ = 1.14 μM and **b**: *K*_i_^*^ = 0.030 μM).

Enzyme inhibition by the benzyl esters **1** and **2** was tested with the fluorogenic substrate Cbz-Phe-Arg-AMC as described previously [18,30]. First, assays with 20 µM inhibitor concentration were performed. For compounds displaying > 60% inhibition at that concentration, concentration-dependent assays were performed and IC_50_ values as well as detailed kinetic constants (*K*_i_, *K*_i_^*^ and *k*_3/4_) were determined (Table 1 and Table 2). The IC_50_ values (In case of time-dependent inhibition, the steady-state residual enzyme activities (vS) were used for determination of the IC50 values) were determined at different substrate concentrations in order to check for competitive or non-competitive inhibition [32,33]. The values were found to increase linearly with increasing substrate concentrations, indicating competitive inhibition (see exemplarily Figure 1 for inhibition of cathepsin L by benzyl esters **1** and **2**). The respective *K*_i_^(*)^ values (Table 1) were determined from plots of the IC_50_ values against the substrate concentration according to the Cheng–Prusoff relationship (Figure 1) [34].

In order to check the cross-reactivity against serine proteases, we determined the potency of the compounds to inhibit the NS3/NS2B protease of the Dengue virus (DENV PR) [27,28].

For the two benzyl esters **1** and **2**, the highest inhibition was found for cathepsin L with the nitrile substituted quinone **2** (*K*_i_^*^ ca. 30 nM). Both esters also inhibited cathepsin B and rhodesain, however with lower inhibitory potencies compared to cathepsin L. Still, the nitrile derivative **2** exhibited the higher inhibition efficiency. Very low inhibition was found for the DENV PR.

**Figure 2:**
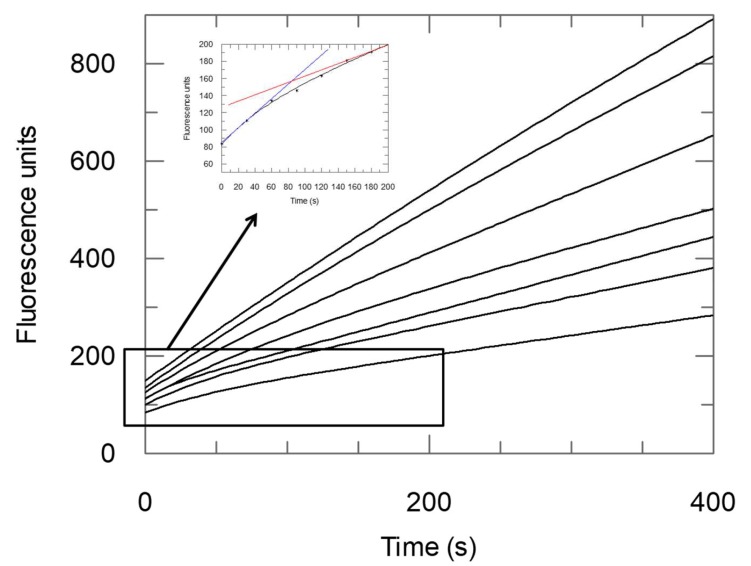
Progress curves for inhibition of cathepsin L by compound **2**. The following inhibitor concentrations were tested (from top to bottom): 0.01–0.05–0.1–0.25–0.5–1.0–2.5 µM. For clarity, the residual initial (v_i_, blue) and the residual steady-state (v_S_, red) velocities are shown exemplarily for the progress curve at [I] = 2.5 μM.

The progress curves for inhibition of cathepsin L were found to be non-linear in case of the nitrile-substituted benzyl ester **2**, i.e., time-dependent inhibition was observed (see Figure 2).

Non-linear progress curves indicate a covalent inhibition mechanism (resulting either from covalent reversible or covalent irreversible inhibition), or indicate a slow/tight-binding non-covalent reversible inhibition. In case of an irreversible inhibition, the progress curves should reach a plateau value with the terminal residual enzyme activity, i.e., the steady-state velocity of substrate turnover in the presence of the inhibitor, v_S_ = 0. In the case of reversible inhibition, the progress curves may also reflect a time-dependent inhibition if a two-step mechanism of inhibition occurs. In these cases, the second step reflects the reversible covalent reaction between a nucleophilic amino acid of the enzyme and the electrophilic warhead of the inhibitor or it reflects the inhibition due to a non-covalent high-affinity complex, e.g., resulting from large conformational changes. In such cases, the progress curves display a biphasic behavior with the terminal residual enzyme activity in the presence of the inhibitor v_S_ ≠ 0, but with v_S_ < v_i_ (with v_i_ being the initial residual enzyme activity in the presence of the inhibitor, Figure 2). The two scenarios, i.e., irreversible and reversible two-step mechanism, may not be distinguished easily when only the progress curves are regarded, requiring additional investigations of the (ir)reversibility of the inhibition, e.g., by dialysis or dilution assays [35].

**Figure 3:**
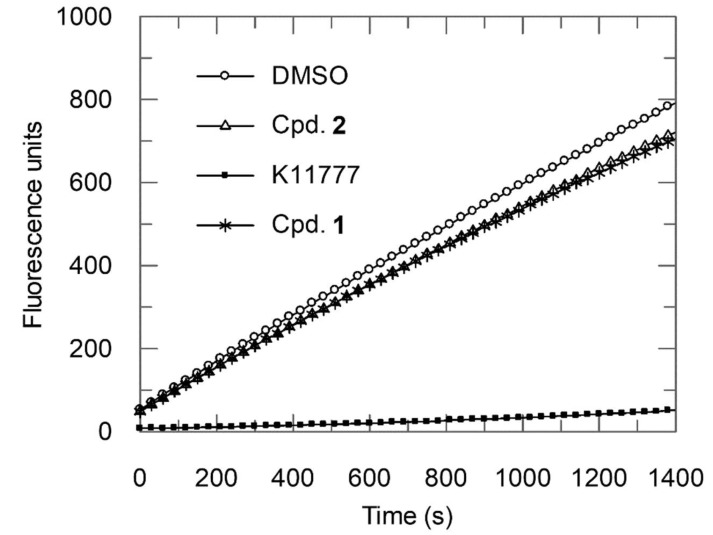
Dilution assays to prove reversible inhibition. Cathepsin L was incubated (15 min) with an excess of inhibitor **1** or **2**, respectively (five times the IC_50_ concentration) to ensure full inhibition. Then, the mixture was diluted by 50-fold to yield a final inhibitor concentration of 10% of the IC_50_ concentration. The enzyme activity, measured through addition of the substrate, nearly fully recovered in case of inhibitors **1** and **2**, whereas in case of the irreversible inhibitor K11777, a well-known vinyl sulfone [36], no recovery was observed.

In order to verify the (ir)reversibility of inhibition, we thus performed dilution assays with cathepsin L and both inhibitors **1** and **2**, as well with rhodesain and chloro-derivative **1**. These assays showed full reactivation of the enzyme activity in the case of cathepsin L for both inhibitors **1** and **2** (see Figure 3), but only partial reactivation (40–50%) in the case of rhodesain and the chloro-derivative **1** (data not shown).

The biphasic behavior observed in the progress curves for inhibition of cathepsin L by compound **2** (Figure 2) with the steady-state velocity of substrate turnover v_S_ ≠ 0, indicates a reversible two-step mechanism, which was supported by the dilution assays. We calculated the dissociation constants of the initial non-covalent enzyme-inhibitor complex *K*_i_ (=$\frac{k_{2}}{k_{1}})$ and of the final, high-affinity complex *K*_i_^*^.

$$\underset{K_{i}^{*}}{\underset{︸}{\underset{k_{i}}{\underset{︸}{E+I\overset{k_{1}}{\underset{k_{2}}{⇌}}E}}---I\overset{k_{3}}{\underset{k_{4}}{⇌}}E-I}}$$

To this end, we determined the initial and steady-state velocities (v_i_ and v_S_) in the presence of the inhibitor (Figure 2) as well as the pseudo-first order rate constants *k*_obs_ of inhibition for seven different inhibitor concentrations [I] by fitting the progress curves (Figure 2) to Equation (1) [37,38]:

(1) $$\left[P\right]=v_{s}*t+\frac{v_{i}-v_{s}}{k_{obs}}\left[1-\exp\left(-k_{obs}*t\right)\right]+offset$$

**Figure 4:**
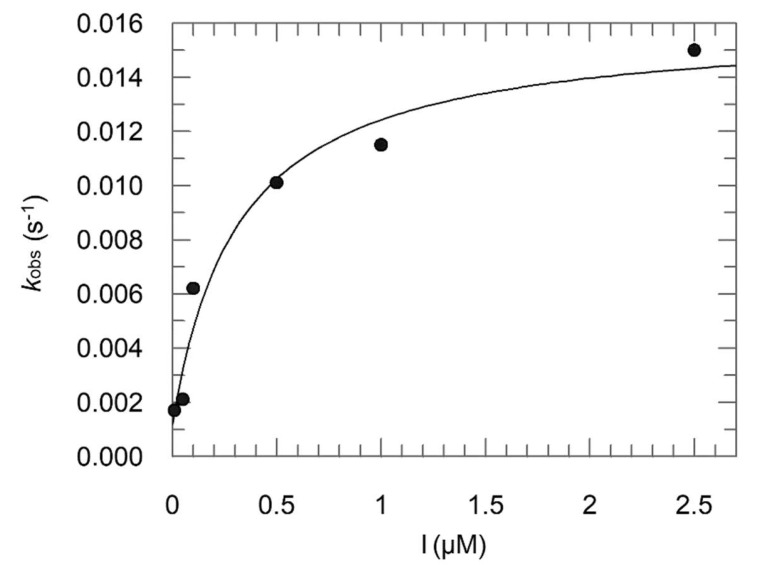
Example of a replot of the *k_obs_* values for the progress curves from Figure 2 as a function of inhibitor concentration. The intercept with the *y*-axis provides an estimate of *k*_4_, while the maximum value of *k_obs_* at infinite inhibitor concentration reflects the sum of *k*_3_ and *k*_4_. The concentration of inhibitor yielding a half-maximal value of *k_obs_* is equal to *K*_i_*^app^*.

The *k_obs_* values were fitted against the inhibitor concentrations [I] (Figure 4) with Equation (2) [37]:

(2) $$k_{obs}=k_{4}+\left(\frac{k_{3}*\left[I\right]}{K_{i}^{app}+\left[I\right]}\right)$$

to yield the apparent dissociation constant *K*_i_*^app^* of the initial enzyme–inhibitor complex, as well as the rate constants *k*_3_ and *k*_4_ of the second inhibition step (method 1).

Since the compound displays competitive inhibition with respect to the substrate (see Figure 1), the *K*_i_*^app^* value could be converted to *K_i_* for the initial inhibitor encounter complex with the Cheng–Prusoff equation (Equation (3)) [34]:

(3) $$K_{i}=\frac{K_{i}^{app}}{1+\frac{\left[S\right]}{K_{m}}}$$

The K_i_^*^ value as the dissociation constant of the final high-affinity complex was calculated from Equation (4):

(4) $$K_{i}^{*}=\frac{K_{i}}{1+\left(\frac{k_{3}}{k_{4}}\right)}$$

Both dissociation constants, *K_i_* and K_i_^*^, were also obtained (method 2) by fitting the initial (*v_i_*) and steady-state (*v_S_*) velocities, respectively, against the inhibitor concentrations using the Dixon equation (Equation (5)) [32,33]:

(5) $$\frac{v_{i,s}}{v_{0}}=\frac{\left[I\right]}{1+\left(\frac{\left[I\right]}{K_{i}^{\left(*\right)app}}\right)}$$

In order to obtain *K_i_^app^* (from fitting *v_i_* against [I]) and K_i_^*app^ (from fitting vs. against [I]) with *v*_0_ as the substrate turnover velocity in the absence of the inhibitor. The apparent dissociation constants were again converted into the true dissociation constant using the Cheng–Prusoff equation (Equation (3)).

The following results were obtained:

Method 1: *K_i_* = 0.16 μM; K_i_^*^ = 0.012 μM; *k*_3_ = 0.0148 s^−1^; *k*_4_ = 0.0012 s^−1^;
Method 2: *K_i_* = 0.30 μM; K_i_^*^ = 0.054 μM.

The results show, that there is good conformity between the two methods for determination of *K_i_* and K_i_^*^ values. The data show that compound **2** displays a two-step inhibition mechanism on cathepsin L with a high affinity (0.012 µM (method 1)/0.054 µM (method 2); i.e., ca. 0.03 µM as mean value) of the final complex and a half-life t_1/2_ of ca. 570 s, i.e., around 10 min(calculated by $t_{1/2}=\frac{0.693}{k_{4}}$).

Assuming that the forward and the backward reaction proceed along the same reaction path and that their kinetic prefactors are equal, the quotient of *k*_3_ and *k*_4_ can be used to estimate the reaction energy Δ*E_reac_* of the second inhibition step by Equation (7), which is derived from Equation (6):

(6) $$\frac{k_{3}}{k_{4}}=\frac{A_{3}\exp\left(-\frac{\Delta E_{3}^{A}}{RT}\right)}{A_{4}\exp\left(-\frac{\Delta E_{4}^{A}}{RT}\right)}=\exp\left(\frac{\Delta E_{4}^{A}-\Delta E_{3}^{A}}{RT}\right)=\exp\left(\frac{\Delta E_{reac}^{}}{RT}\right)$$

(7) $$\Delta E_{reac}^{}=ln\left(\frac{k_{3}}{k_{4}}\right)RT$$

In Equation (6), $\Delta E_{3}^{A}\;\mathrm{and}\;\Delta E_{4}^{A}$ are the corresponding reaction barriers of the second inhibition step; their difference corresponds to the reaction energy Δ*E_reac_* of this step. Using the *k*_3_ and *k*_4_ values measured for **2**, a reaction energy Δ*E_reac_* of about −1.5 kcal/mol was obtained.

### 2.3. Mass Spectrometry with Benzyl Esters **1** and **2**

**Figure 5:**
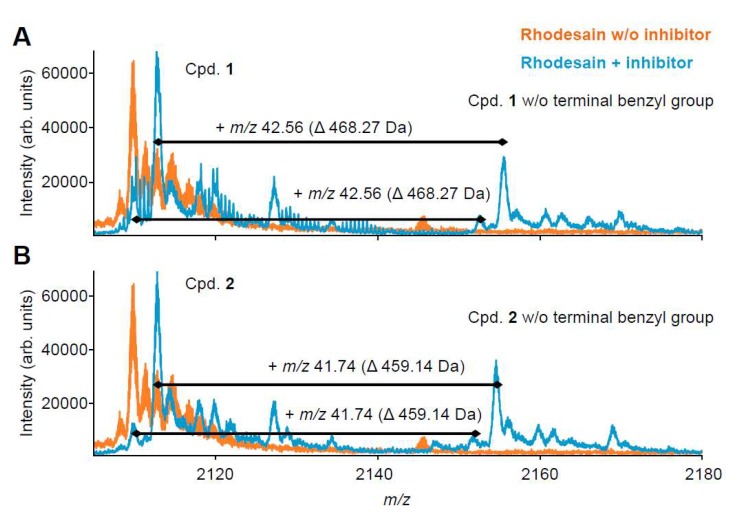
ESI–MS (electrospray ionization) mass spectra (MS) of rhodesain ([M + 11 H]^11+^ at *m/z* 2109.9 ± 20 ppm) in the absence (orange) or presence (blue) of compounds **1** (**A**) and **2** (**B**). The addition of compounds **1** and **2** resulted in mass shifts of (**A**) *m/z* 42.56 (corresponding to 468.27 Da) and (**B**) *m/z* 41.74 (corresponding to 459.14 Da), in both cases indicating the formation of an adduct between rhodesain and the hydrolysis product (i.e., the acid) of the respective compound.

To further characterize the interaction between rhodesain and the two benzyl ester-based inhibitors **1** and **2**, we performed a liquid-chromatography–mass spectrometric (LC–MS) analysis of rhodesain that had been incubated with the compounds. Rhodesain without an inhibitor served as a control. Both compounds reacted with rhodesain (Figure 5).

Interestingly, for both investigated compounds we found that rhodesain catalyzed the hydrolysis of the benzyl ester of the dipeptide recognition unit to the corresponding acid, indicated by a mass shift of 90 Da corresponding to the loss of the terminal benzyl group (Figure 5). Notably, only adducts of the hydrolysis products (i.e., the acids) with rhodesain were detectable by LC–MS analysis. This is in full agreement with previous results, which also revealed enzyme-catalyzed hydrolysis of peptidic benzyl esters with electrophilic warhead by rhodesain yielding free acids as highly active inhibitors [30].

### 2.4. Enyzme Assays with Acids **3** and **4**, and Esters **5**–**8**

In order to investigate whether this inhibition mode is also found for other esters, we synthesized and tested the respective methyl and *tert*-butyl esters **5**–**8**. None of these compounds was found to significantly inhibit either cathepsin L or rhodesain at 20 µM.

The free acids **3** and **4**, which were identified via mass spectrometry as rhodesain inhibitors emerging through hydrolysis of the benzyl esters by the target enzyme, were synthesized and tested. While in the case of cathepsin L, we observed a 20-fold improvement for the chloro-derivative **3** compared to the ester **1** (1140 vs. 57 nM), the nitrile-substituted acid **4** turned out to be comparable in potency to its benzyl ester **2** (33 vs. 17 nM). In agreement with previous findings [30], the inhibition of rhodesain was extraordinarily improved with the two acid derivatives **3** and **4** (with 2900 vs. 24 nM ca. 120-fold for the chloro-derivative **3** compared to the ester **1**, with 180 vs. 0.15 nM ca. 1200-fold for the nitrile derivative **4** compared to the ester **2**). For cathepsin B and the DENV PR no improvement in inhibition was observed.

**Figure 6:**
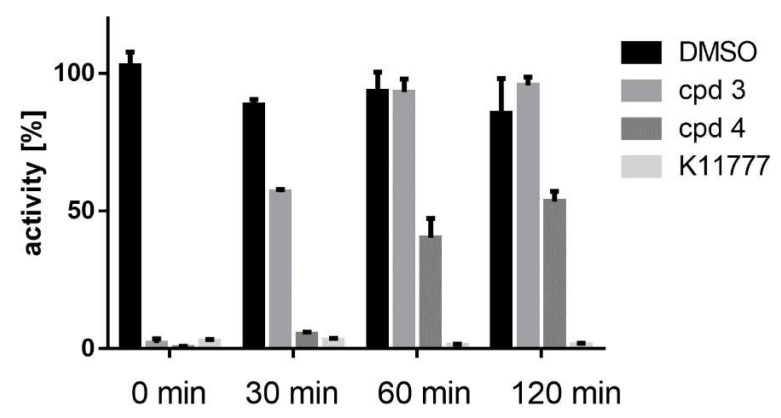
Dialysis assay to investigate the reversibility of the inhibition. Rhodesain was preincubated (30 min) with inhibitor **3**, **4** or K11777, respectively, (20 times the IC_50_ concentration). Then, the mixture was dialyzed in a self-made dialysis chamber [35]. The enzyme activity of all samples was determined relatively to the DMSO treated sample (0 min) by a fluorometric enzyme assay.

Dilution assays with the five-fold IC_50_ concentrations and 50-fold dilution for the carboxylic acid inhibitors **3** and **4** were performed on both, cathepsin L and rhodesain. These assays showed full reactivation of the enzyme activity for both inhibitors on both enzymes (data not shown). To strengthen the statement of reversible inhibition, we performed dialysis assays with the 20-fold concentration of the IC_50_ of inhibitor **3** or **4** and rhodesain (Figure 6). For both compounds, **3** and **4**, the enzymatic activity was restored by dialyzing the inhibitor from the reaction vessel. In the case of compound **4**, i.e., the highly potent nitrile-derivative, only slow reactivation was observed. In the case of the known irreversible inhibitor K11777 [36,39], the enzyme activity could not be recovered.

**Figure 7:**
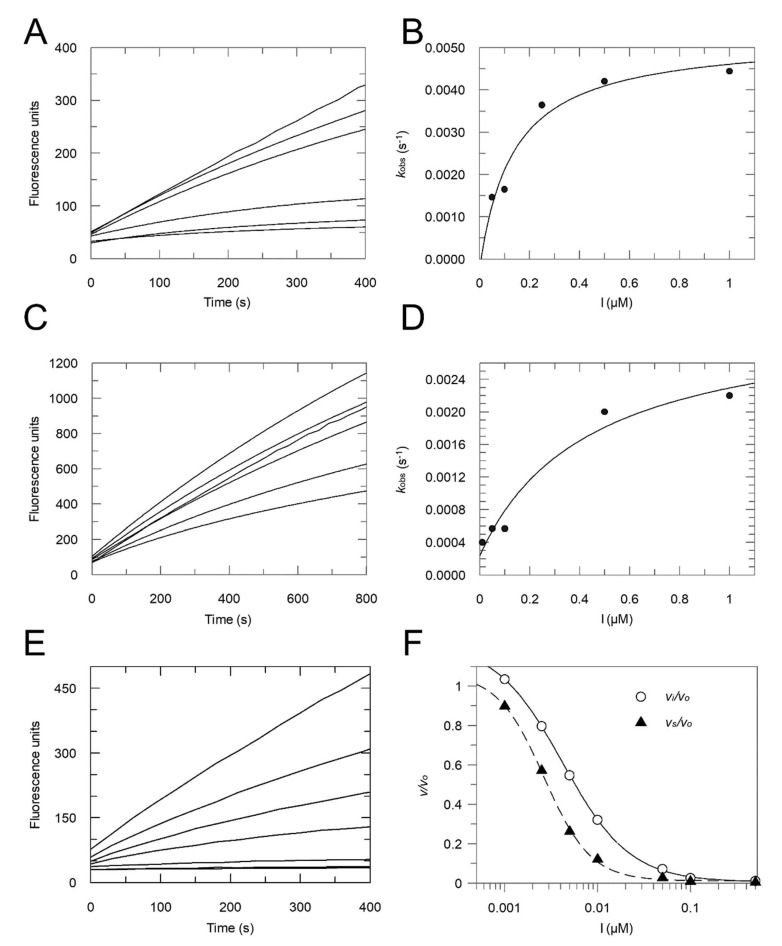
Inhibition of cathepsin L by compound **4** (**A**,**B**), rhodesain by compound **3** (**C**,**D**) and rhodesain by compound **4** (**E**,**F**). **A**,**C**,**E**: Progress curves with the following inhibitor concentrations (each from top to bottom): 0–0.05–0.1–0.25–0.5–1.0 µM (**A**); 0–0.01–0.05–0.1–0.5–1.0 µM (**C**) and 0.001–0.0025–0.005–0.01–0.05–0.1–0.5 µM (**E**). **B, D:** Replots of the *k_obs_* values for the progress curves from Figure **7A** (→**B**) and **7C** (→**D**) as a function of inhibitor concentrations. The intercept with the *y*-axis provides an estimate of *k*_4_, while the maximum value of *k_obs_* at infinite inhibitor concentration reflects the sum of *k*_3_ and *k*_4_. The concentration of inhibitor yielding a half-maximal value of *k_obs_* is equal to *K_i_^app^*. **F**: Plot of the v_i_/v_o_ and v_S_/v_o_ values obtained from the progress curves of plot E as a function of inhibitor concentrations to yield *K_i_^app^* and K_i_^*app^, respectively.

Both acids were found to inhibit both enzymes, cathepsin L and rhodesain, reversibly (Figure 6), but time-dependently (Figure 7A,C,E; see Table 1 and Table 2 for detailed kinetic data). Dissociation constants *K_i_* and K_i_^*^, the rate constants *k*_3_ and *k*_4_ and the half-lives t_1/2_ and reaction energies Δ*E_reac_* were obtained as described above (see Table 2, see exemplarily Figure 7 for inhibition of cathepsin L by compound **4**, and rhodesain by compounds **3** and **4**).

The detailed kinetic data for the two acid derivatives show that the *k*_3_ and *k*_4_ values, and as a consequence also t_1/2_ and Δ*E_reac_* were in the same range for all tested systems. In general, the high overall inhibition exhibited by the compounds was mainly due to very low off-rates *k*_4_ leading to relatively long half-lives (770–1155 s, i.e., 10–20 min). The differences in the overall inhibition potencies of the two acid derivatives against the two enzymes (represented by the *K*_i_^*^ values) mainly originated from the different stabilities of the initial non-covalent complexes [E−−−I], represented by the *K*_i_ values (ca. 400 nM for **3**-CathL; ca. 100 nM for **4**-CathL; ca. 60 nM for **3**-Rhod and ca. 0.4 nM for **4**-Rhod). Thus, the different overall potencies were mainly due to the non-covalent interactions and not to the covalent second inhibition steps. This is especially apparent for the very strong inhibition of rhodesain by compound **4**: the *k*_3_ and *k*_4_ values for the second, covalent step were similar to those of the other enzyme-inhibitor pairs, but the dissociation constant for the initial inhibition step was much lower (*K*_i_ ca. 0.4 nM) than those of the others indicating an extremely stable initial non-covalent complex [E−−−I] finally leading to an extraordinarily strong overall inhibition (K_i_^*^ ca. 0.15 nM).

### 2.5. Hydrolysis Assays with Benzyl, Methyl and tert-Butyl Esters

As reported previously for benzyl esters of the tested dipeptide sequence [30], we raised the hypothesis also in the current case, that the observed ester cleavage of the rhodesain–inhibitor adduct (Figure 5) might occur by rhodesain-catalyzed enzymatic hydrolysis. To verify this hypothesis, we incubated compounds **1**, **2** (benzyl esters), compound **6** (methyl ester) and compound **8** (*tert*-butyl ester) for 24 h with rhodesain. The reaction mixtures were subsequently analyzed by LC–MS.

**Figure 8:**
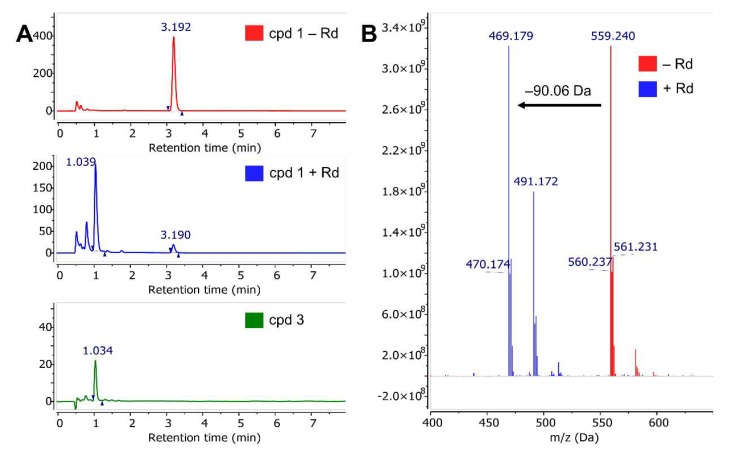
Hydrolysis assay to investigate the ester hydrolysis prodrug concept. (**A**) Exemplarily, the chromatograms for cpd. **1** are shown. Cpd. **1** hydrolyzed with rhodesain (blue, t_R_ = 1.03 min) shows the same retention time as the reference cpd. **3** (green, t_R_ = 1.03 min). The negative control incubated without rhodesain shows only the educt peak of cpd. **1** (red, t_R_ = 3.19 min). (**B**) Analysis of the mass spectrum reveals the loss of a 90 Da fragment, matching the mass and isotopic distribution of the predicted enzymatic benzyl ester hydrolysis ([cpd**1** + H]^+^ = 559.2 m/z; [cpd**3** + H]^+^ = 469.2 m/z).

In fact, we found ester hydrolysis for the benzyl and methyl esters, but not for the *tert*-butyl ester. Negative controls without rhodesain showed that the esters were not hydrolyzed by the reaction buffer. Exemplarily, the LC–MS analysis of compound **1** is shown in Figure 8. The unconverted ester **1** was eluted at t_R_ = 3.19 min. Upon incubation with rhodesain, a peak at t_R_ = 1.03 min was observed matching the retention time of the reference acid **3**. The detected masses matched the theoretical 90 Da loss of a benzyl group ([cpd**1** + H]^+^ = 559.2 m/z; [cpd**3** + H]^+^ = 469.2 m/z) and the isotopic pattern for the chlorine substituent demonstrating the rhodesain-catalyzed ester hydrolysis.

These findings again support our previous identification of the benzyl ester prodrug concept for rhodesain inhibitors [30]: dipeptide-derived benzyl esters containing an electrophilic building block were hydrolyzed by the target enzyme itself to yield the free acids as highly potent inhibitors. According to the MS data, which show the *m/z* peak of the adduct of the enzyme and the complete free acid, the inhibition proceeds via a nucleophilic addition reaction rather than a combined addition/elimination (i.e., a substitution) reaction under the loss of chloride or cyanide. This is in agreement with previous studies on halogen-substituted Michael-acceptors, which showed slow elimination of the halide only in the case of bromine [17].

**Figure 9:**
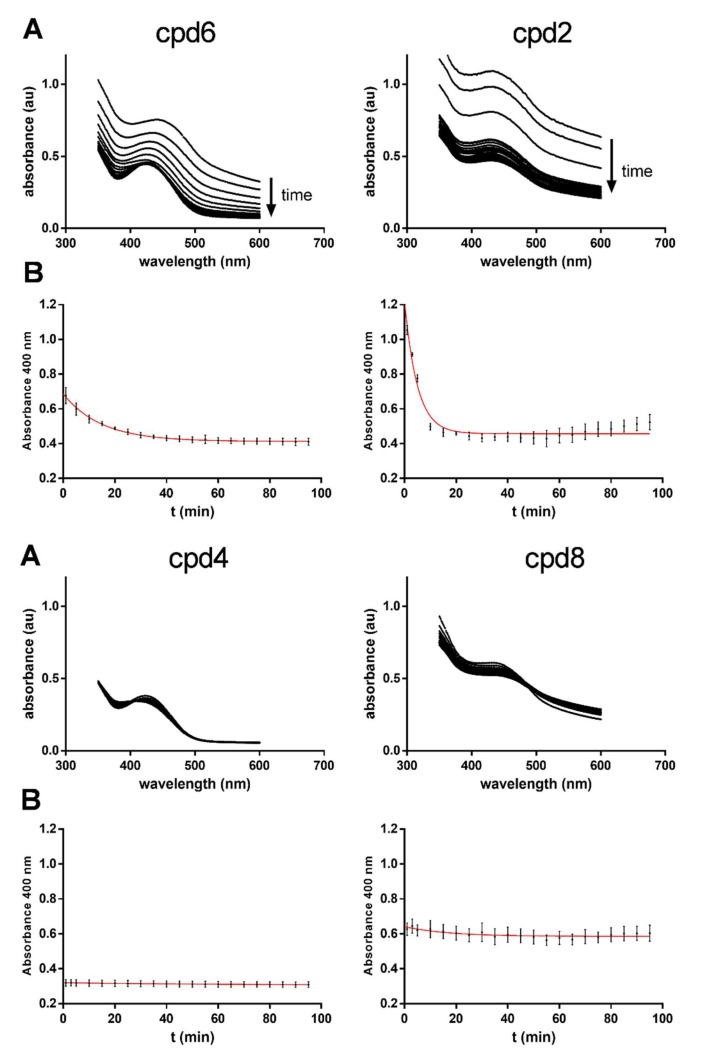
Kinetic analysis of the ester hydrolysis by rhodesain. (**A**) Time-dependent measurement of absorbance spectra (λ = 350–600 nm) of the respective compounds treated with rhodesain; (**B**) Plot of absorbance at 400 nm versus time showed hyperbolic turnover curves for the methyl ester (cpd. **6**, t_1/2_ = 10.2 min) and the benzyl ester (cpd. **2**, t_1/2_ = 3.41 min) but not for the free acid (cpd. **4**) and the *tert*-butyl ester (cpd. **8**).

In order to determine whether the differences in activity between the benzyl esters and the methyl ester are due to different rates of hydrolysis catalyzed by rhodesain yielding the acid as much more potent inhibitor, we followed the reactions spectrophotometrically. Since the esters and the free carboxylic acid have different UV-Vis characteristics, we recorded time-dependent absorbance spectra at 400 nm (Figure 9). Plots of absorbance at 400 nm vs. time showed hyperbolic turnover curves for the benzyl ester (cpd. **2**) and the methyl ester (cpd. **6**), but not for the *tert*-butyl ester (cpd. **8**) or the free carboxylic acid (cpd. **4**) We were able to show that the benzyl ester (t_1/2_ = 3.41 min) was hydrolyzed about three times faster than the methyl ester (t_1/2_ = 10.2 min), which supports the hypothesis that in-situ hydrolysis of the benzyl ester to the more active acid may in parts also be responsible for its inhibitory activity.

### 2.6. Reaction of Benzyl Esters **1** and **2** with a Low Molecular Weight Thiol

**Scheme 2:**
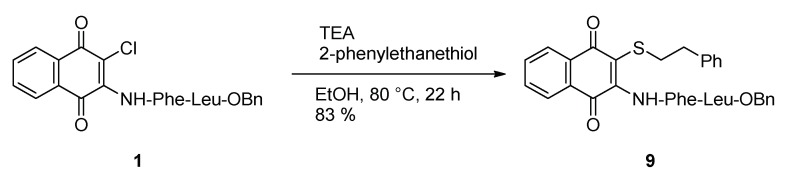
Substitution reaction with 2-phenylethanethiol.

To further investigate the interaction between the inhibitors and a low molecular weight thiol (LMW thiol), the reactions of the esters **1** and **2** with 2-phenylethanethiol in the presence of a base in ethanol were carried out. It is worth mentioning that, with excess thiol and triethylamine at 80 °C, inhibitor **1** gave the substitution product **9** (Scheme 2) with thiol, which—from a chemical point of view—could be expected (yield 83%), while inhibitor **2** did not react with the thiol at all under the same reaction conditions. These results were in contrast to the reactions of the inhibitors within the enzyme, which yielded the addition products with the chloro- and the nitrile-substituted inhibitor, but not the substitution products (see below).

### 2.7. Quantum-Mechanical Computations

**Scheme 3:**
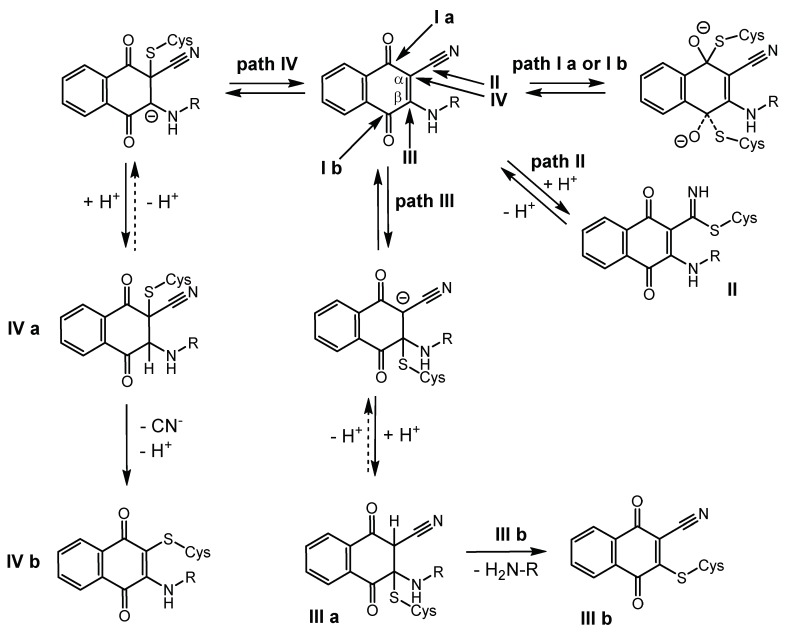
Possible reaction mechanisms of the 1,4-naphthoquinones with the active-site cysteinate residue, elucidated by quantum-chemical computations. For chloro-substituted compounds, the same pathways and products, except **II**, should be possible.

All tested compounds contain an activated double bond that could undergo a nucleophilic addition or substitution reaction at one of the C-atoms of the α,β-unsaturated carbonyl function with the Cys residue of the active site of cysteine proteases. According to the MS studies, an addition reaction is displayed by both acids **3** and **4**. For unsubstituted naphthoquinones [22], the attack at the β-C atom was proposed. It is known from kinase inhibitors [40] and also from cysteine protease inhibitors [17] that Michael-acceptor systems can be switched from irreversible nucleophilic addition to reversible addition by introduction of an electron-withdrawing group X (X = halogen, nitrile) at the α-position [41]. A reversible addition is also observed for the inhibitors presented herein. As discussed above, in some cases (X = Br), slow irreversible inactivation was found with such inhibitors due to release of the halide anion [17]. On the other hand, nitrile groups are well-known to undergo covalent reversible addition reactions with the active site Cys or Ser residues of cysteine and serine proteases to yield a thioimidate and imidate, respectively [18,19,20,21]. Taken together, the structures of the compounds provide several possibilities (Scheme 3) for reactions with the nucleophilic active site Cys residue: reversible nucleophilic attack at one of the carbonyl carbon atoms (Scheme 3: **I a,b**), reversible attack at the nitrile group (**II**) or attack at one of the C-atoms of the double bond (**III, IV**). From the reversible attack at the nitrile group, compound **II** results after protonation. In the case of the β-attack (**III**), the reaction should be reversible since an *S*,*N*-acetal is produced and elimination of the amino group (yielding **III b**) should not be favored, even after its protonation. In case of the α-attack (**IV**), the addition reaction could be either reversible or irreversible (**IV a**). If an elimination of cyanide (or chloride, not shown in the Scheme 3) occurs (**IV b**) the reaction will become irreversible. For the chloro-derivatives, the corresponding products, except **II**, should be possible. According to the MS studies, addition reactions take place within the enzyme for both, the nitrile- and the chloro-substituted compounds. However, the obvious differences between the reaction with an LMW thiol in solution and the reaction within the enzyme have to be considered: with an LMW thiol, the substitution reaction takes place with the chloro-substituted ester (path **IV b**), while the nitrile compound is virtually unreactive.

In order to shed some light on possible inhibition mechanisms, we performed quantum-mechanical (QM) computations of the reaction energies of all possible reactions. In these simulations, the HN-Phe-Leu-OR/OH group was replaced by HNCH_3_ and the attacking cysteine group was mimicked by methanethiol. In the computations, the influence of water was taken into account through the conductor-like polarizable continuum model (C-PCM) approach [42]. The calculations were performed with the Gaussian 16 program packages [43]. Our computations only focus on reaction energies of the reactions in solvents, i.e., kinetic effects, the influence of the enzyme environment and entropy effects are neglected, which all may influence which reaction path is taken. However, comparable calculations were previously successfully employed to get insights into the inhibition mechanisms of various electrophilic warheads such as three-membered heterocycles [44,45,46] and Michael-systems [47]. Additionally, more detailed simulations that compute the whole reaction path, take the molecular nature of the enzyme environment and entropy effects into account and thus can provide quantitative predictions about the kinetics of the reactions [48,49] are hardly feasible in the present case because experimental information about the orientation of the inhibitor within the active site of the enzymes are missing. Table 3 shows possible products and intermediates resulting from the various reaction paths given in Scheme 3. Please note that **III a 1** and **III a 4** are enantiomers. The same holds for **III a 2** and **III a 3**; **IV a 1** and **IV a 4** as well as **IV a 2** and **IV a 3**. Nevertheless, we present all data to show that our computations indeed found the same conformers in each case. To ensure that we computed the lowest conformers we characterized various additional conformers, which were all higher in energy.

The experimentally determined reversibility of the inhibition for both, the benzyl esters and the acids is in line with a reversible addition to the double bond because backward reactions from the intermediates **I**, **III a** and **IV a** are possible if the exothermicity of the addition reaction is small. The same holds true for the formation of **II,** which represents the product of the addition to the CN group. Such a reaction is also known to be reversible. In contrast, backward reactions from the intermediates **III b** and **IV b** are unlikely because the corresponding leaving groups cyanide (CN^−^), chloride (Cl^−^) or H_2_N-R are not expected to reattack the double bond. Their reactivity is low and they will diffuse away from the reaction site after the bond dissociation. Hence, the reaction paths **III b** and **IV b** would not be in line with the experimental findings concerning the reaction within the enzyme, which neither yielded substitution products with the enzyme for the chloro- nor for the nitrile-substituted derivatives. However, such a reaction path is found for the chloro-derivative in reaction with a low molecular weight thiol. Thus, these steps should be included in the simulation because an insight into the relative energetics of all reaction paths answers the question if the inhibition mechanism might be steered by substituent effects.

The computed data for the nitrile are summarized in Table 4 (C-PCM with ε = 78.36). DFT approaches are known to overestimate stabilization effects in delocalized systems. Consequently, DFT might considerably overestimate the stability of the reactants and of **II**, **III b** and **IV b** with respect to the intermediates **III a 1-4** and **IV a 1-4**, i.e., addition-elimination mechanisms would be erroneously favored with respect to simple addition reactions. Hence, to obtain insights into the applicability of DFT functionals we compared the predictions of B3LYP-D [50,51], CAM-B3LYP, CAM-B3LYP-D [52], M06, M06-2X [53] and ωB97-XD [54] with the computed values of the spin component scaled Møller–Plesset perturbation theory second order (SCS-MP2) approach [55,56]. For the present model computations, we focus on reaction energies (Δ*E_reac_*) rather than on free reaction energies (Δ*G_reac_*) since the entropy effects are more important for the formation of the non-covalent reversible enzyme-inhibitor complex, which represents a bimolecular reaction. The chemical reaction between inhibitor and enzyme, which will take place after the formation of this complex, represents a unimolecular reaction, i.e., entropy effects are expected to be small within this step.

According to the presumably most accurate SCS-MP2 approach, the formation of **III a 1** and its enantiomeric form **III a 4** were mostly exothermic (Δ*E_reac_* = −8.5 kcal/mol). **III a 2** and its enantiomeric form **III a 3** were predicted to be less stable. For the attack to the nitrile group (formation of **II**), a reaction energy of −1.8 kcal/mol was computed. The most stable isomers of compounds **IV a** were **IV a 1** and **IV a 4** with about −5 kcal/mol. The compounds from which no back-reaction was expected (**III b**, **IV b**) were computed to be very unstable (+9.4 kcal/mol and +8.8 kcal/mol, respectively). DFT calculations came to different conclusions: All functionals predicted compound **II** to be lower in energy than the addition products connected with **III a** or **IV a**. The computed stabilities of **II** with respect to the reactants varied between −0.1 kcal/mol (CAM-B3LYP) and about −5 kcal/mol (ωB97-XD). Products resulting from the addition of methanethiol to the activated double bond were computed to be considerably less stable, in some cases the corresponding reaction was computed to be endothermic. The difference between the wave function and the DFT-based approaches might result because many DFT functionals often overestimate stabilities of delocalized systems. Such effects stabilized the reactants, **II**, **III b** and **IV b** with respect to **III a** and **IV b**. This again underlines that the DFT-based predictions about the reaction mechanisms of warheads have to be regarded with care if compounds with different degrees of delocalization are involved.

The computations provided insights into the thermodynamics of the possible reaction mechanisms, however effects arising from the enzyme environments were neglected. Endothermic mechanisms were not expected to take place. However, the computations could not predict which of the exothermic reactions did actually take place since this is also dependent on the environment within the enzyme and the geometry of the non-covalent enzyme-inhibitor complex. Computations that take these influences into account, e.g., QM/MM or even QM/MM/MD computations, are only possible when the necessary crystallographic information of the enzyme–inhibitor complex are available, which was not the case for the systems presented herein.

The reaction energies obtained from the measured *k*_3_ and *k*_4_ values for the nitrile-compounds **2** and **4** were between −0.89 and −1.5 kcal/mol. These values fit best to the value for the attack at the nitrile group computed with SCS-MP2 but the formation of **III a** or **IV a** were predicted to be more exothermic, i.e., they should be formed. However, within an enzyme environment they might become less favored due to two reasons. Firstly, the orientation of the inhibitor within the active site might only allow an attack at the nitrile. Additionally, the formation of **III** or **IV** might be hampered by steric effects because the flat sp^2^ hybridized inhibitor is transformed into a considerably more bulky form due to the formation of two sp^3^ centers. Nevertheless, the computations definitely excluded the formation of intermediates, which should lead to an irreversible enzyme inhibition. This was in line with the experimental results. It is also important to note that the formation of **IV b,** which was not observed in the solvent experiment, was definitively excluded by our computations.

The differences between SCS-MP2 and DFT approaches were even more obvious for the chloro-substituted 1,4-naphthoquinone (Table 5). As for the nitrile compound, SCS-MP2 predicts that the formation of compounds **III a 1** and its enantiomer **III a 4** were most favorable (Δ*E_reac_* = −17.3 kcal/mol). Additionally, as computed for the nitrile compound, other compounds could be formed because their reactions energies differed only slightly from those of the formation of **III a 1** and **III a 4**. However, the products that indicate an irreversible substitution reaction (**III b** and **IV b**), were again too high in energy to be formed. In contrast, most DFT functionals predicted the formation of compound **IV b**, i.e., the addition to the double bond followed by the elimination of HCl (H^+^ + Cl^−^ ). Since the elimination product (i.e., chloride) is expected to diffuse away and because it is not sufficiently reactive for a reverse reaction, most functionals predicted an irreversible reaction. This was in agreement with the experimental findings for the reaction of the chloro-derivative with excess LMW thiol in solution in the presence of a base. The product of this solution reaction corresponds to product **IV b** in which the chloride was substituted by the attacking thiol group. Does this result indicate that the DFT functionals are right while SCS-MP2 is wrong? This is not the case. For **III a 1**, SCS-MP2 predicted a reversible reaction, i.e., reactants and products were in equilibrium. The formation of **IV b** was also predicted to be exothermic, i.e., this reaction would also take place. Due to the difference in the exothermicity, **IV b** was formed to a lower extent, but because this reaction is irreversible while the formation of **III a 1** is reversible, finally only the product of the irreversible reaction would be found. For the enzyme environment, both reactions can be expected to be in equilibrium because the chloride as elimination product will be formed within the active site of the enzyme, i.e., its diffusion will be strongly hindered. In this case, the formation of e.g., **III a 1** would be considerably favored so that the irreversible reaction will be suppressed. It is also important to note that our computations are in line with the experimental observation that in the solvent reaction of an LMW thiol with the chloro-substituted compound the elimination product **IV b** was formed while it was not formed for the CN-substituted one. 

For the chloro-substituted compound, the experimental and computed reaction energies differed considerably. This may be owed to the fact that the computations neglected steric as well as electronic effects arose from the enzyme environment.

According to the QM computations, for both compound classes, namely the chloro and the nitrile derivatives, the reversible attack of the thiolate at the α- or β-position of the double bond (yielding products **III a** or **IV a**) should be possible. The reaction with a LMW thiol took place at the α-C atom in case of the chloro derivative (yielding **IV b**). The experimentally determined Δ*E_reac_* values for both the chloro and the nitrile derivatives (**2**–**4**) were in the same range for both enzymes (−0.85 to −1.5 kcal/mol, see Table 2). Taken together, it may be hypothesized that for at least the chloro-substituted acid, the second covalent inhibition step is the reversible addition of the active-site Cys residue at the α-position, and furthermore, that this inhibition mechanism might also hold true for the nitrile-substituted derivatives.

### 2.8. T. b. brucei Cell Survival Assay

Benzyl ester **2** was tested for its antitrypanosomal activity as described previously [59]. With an EC_50_ value of 0.12 (±0.013) µM (48 h), the compound was found to exhibit very high antitrypanosomal activity. This finding is in agreement with previous investigations, which yielded benzyl esters of the same dipeptidic recognition unit as highly active antitrypanosomal compounds [30]. Again, our results underlined the hypotheses that the benzyl ester **2** is a prodrug with good cell permeability properties that is hydrolyzed by its target enzyme yielding the free acid as highly active rhodesain inhibitor.

## 3. Discussion

In summary, we presented the facile synthesis and detailed investigation of a class of highly potent protease inhibitors based on 1,4-naphthoquinones carrying a peptidic recognition motif in the 2-position and an electron-withdrawing substituent in the 3-position. A member of this class, namely the nitrile-substituted compound containing the dipeptide sequence NH-l-Phe-l-Leu-OH, was shown to be a highly potent inhibitor of rhodesain, an essential protease of a human-pathogenic parasite, with subnanomolar affinity. To investigate the exact mode of inhibition exerted by this compound class, detailed kinetic and mass spectrometry studies were used and revealed a time-dependent, reversible-covalent binding mode. This particular mode is highly attractive in providing long residence times while at the same time suppressing undesired off-target activity. As found in our previous studies, which reported inhibitors with the same peptidic recognition unit [30], the respective benzyl ester is hydrolyzed by the target enzyme itself yielding the free acid. To better understand the physicochemical basis of the involved processes, we performed calculations with different DFT functionals as well as wave function-based approaches. These were compared in an extensive analysis in which the SCS-MP2 method, but not the investigated density functional turned out to provide reliable estimates for the energetics of the possible individual reaction steps. The knowledge gained in this multifaceted study provides an early step towards the goal of the rational design of new covalent-reversible electrophilic warheads for enzyme inhibitors. The latter ideally provide desirable pharmacokinetic parameters and may even confer resistance-breaking properties as already demonstrated for “simple” covalent kinase inhibitors applied in human oncotherapy.

## 4. Materials and Methods 

See Supplementary Materials.

## Figures and Tables

**Table 1:** Inhibition data for 1,4-naphthoquinone (NQ)-derived inhibitors of the general structure NQ-NH-l-Phe-l-Leu-OR, % inhibition at 20 µM or *K*_i_^(*)^ (nM) ^1^.

Cpd.	Cath. L % inh.	Cath. L *K*_i_^(*)^	Cath. B % inh.	Cath. B *K*_i_^(*)^	Rhod. % inh.	Rhod. *K*_i_^(*)^	DENV PR % inh.	DENV PR *K*_i_^(*)^
**1**		1140	53%			2900	41%	Nd
**2**		33 ^3^		1900		180		41,000 ^2^
**3**		57 ^3^	25%	Nd		24 ^3^	43%	Nd
**4**		17 ^3^	45%	Nd		0.15 ^3^	38%	Nd
**5**	45%	Nd	Nd	Nd	48%	Nd	Nd	Nd
**6**	40%	Nd	Nd	Nd		1235	Nd	Nd
**7**	38%	Nd	Nd	Nd	51%	Nd	Nd	Nd
**8**	21%	Nd	Nd	Nd	38%	Nd	Nd	Nd

^1^ All values are from at least three independent measurements; standard deviations are 15% or less in case of *K*_i_ values and 5% or less in case of % inhibition @ 20 µM; IC_50_ values and *K*_i_^(*)^ values were determined for compounds displaying > 60% inhibition at 20 µM; ^2^ IC_50_ value in nM at [S] = 100 µM (Boc-Gly-Arg-Arg-AMC); ^3^ reversible, time-dependent inhibition; the *K*_i_^*^ values for the final high-affinity complexes are given as mean values from method 1 and method 2 (see Section 2.2); for more detailed inhibition constants see Table 2. Cpd., compound; Cath. L, cathepsin L; Cath. B, cathepsin B; Rhod., rhodesain; DENV PR, Dengue virus protease. Nd, not determined.

**Table 2:** Detailed inhibition data for the time-dependent inhibition of cathepsin L and rhodesain by 1,4-naphthoquinone (NQ)-derived inhibitors of the general structure NQ-NH-l-Phe-l-Leu-OR ^1^.^.^

Cpd.	Cath. L				Rhod.			
	*K_i_*/K_i_^*^ Method 1	*K_i_*/K_i_^*^ Method 2	*k*_3_/*k*_4_	t_1/2_/ Δ*E_reac_*	*K_i_*/K_i_^*^ Method 1	*K_i_*/K_i_^*^ Method 2	*k*_3_/*k*_4_	t_1/2_/ Δ*E_reac_*
**2**	160/12	300/54	0.0148/0.0012	570/ −1.5	Nd	Nd	Nd	Nd
**3**	443/53	345/61	0.0052/ 0.0007	990/ −1.2	57/11	63/36	0.0025/ 0.0006	1155/−0.85
**4**	129/19	91/15	0.0051/ 0.0009	770/ −1.1	0.47/0.10	0.32/0.20	0.0027/ 0.0006	1155/−0.89

^1^*K_i_*^(*)^ values in nM; *k*_3_, *k*_4_ in 1/s, t_1/2_ in s, Δ*E_reac_* in kcal/mol; values are mean values from at least five independent measurements with standard deviations 15% or less. Nd, not determined.

**Table 3:** Reactants and products of the reaction of methanethiol with the 1,4-naphthoquinones. The corresponding products (except **II**) for the chloro-derivatives are obtained by replacing CN by Cl.

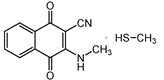			
**reactants**			
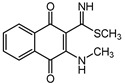			
**II**			
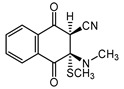	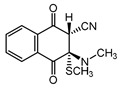	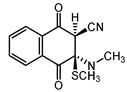	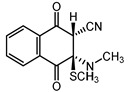
**III a 1**	**III a 2**	**III a 3**	**III a 4**
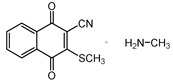			
**III b**			
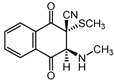	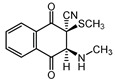	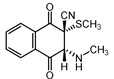	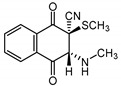
**IV a 1**	**IV a 2**	**IV a 3**	**IV a 4**
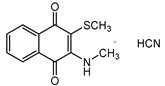			
**IV b**			

**Table 4:** Summary of the reaction energies (Δ*E_reac_*) of possible reaction products for the reaction of the nitrile derivative with methanethiol (Scheme 3, Table 3). All calculations were performed with 6-311++G(d,p) basis sets [57] in combination with the C-PCM approach (solvent = water) to mimic environmental effects. The structures were previously optimized with ωB97-XD/6-311G(d,p) [58]. All energy values in kcal/mol.

Method	SCS-MP2	B3LYP-D	CAM-B3LYP	CAM-B3LYP-D	M06	M06-2X	ωB97-XD
**II**	−1.8	−2.8	−0.1	−3.7	−4.5	−4.7	−4.9
**III a 1**	−8.5	5.5	8.2	2.1	1.1	−1.8	1.2
**III a 2**	−6.2	6.5	8.9	2.8	1.6	−1.3	2.1
**III a 3**	−6.2	6.5	8.9	2.8	1.6	−1.3	2.1
**III a 4**	−8.5	5.5	8.2	2.1	1.1	−1.8	1.2
**III b**	9.4	15.7	17.3	17.2	15.1	15.2	16.9
**IV a 1**	−5.2	9.8	12.0	6.3	6.0	2.8	5.6
**IV a 2**	−1.6	14.0	15.6	10.4	10.3	6.4	9.3
**IV a 3**	−1.6	14.0	15.6	10.4	10.3	6.4	9.3
**IV a 4**	−5.2	9.8	12.0	6.3	6.0	2.8	5.6
**IV b**	8.8	14.0	15.2	13.8	13.7	11.5	13.9
**MAE ^1^**		**11.6**	**13.5**	**9.2**	**8.6**	**6.1**	**8.6**

^1^ Mean average error with respect to SCS-MP2 averaged of all compounds.

**Table 5:** Summary of the relative energies of possible reaction products for the reaction of the chloro-derivative with methanethiol (Scheme 3, Table 3). All calculations were performed with 6-311++G(d,p) basis sets in combination with the C-PCM approach (solvent = water) to mimic environmental effects. The structures were previously optimized with ωB97-XD/6-311G(d,p). All energy values were in kcal/mol.

Method	SCS-MP2	B3LYP-D	CAM-B3LYP	CAM-B3LYP-D	M06	M06-2X	ωB97-XD
**III a 1**	−17.3	−4.6	−1.3	−7.8	−9.0	−11.4	−8.3
**III a 2**	−13.2	−1.0	1.4	−4.7	−5.7	−8.7	−5.3
**III a 3**	−13.2	−1.0	1.4	−4.7	−5.7	−8.7	−5.3
**III a 4**	−17.3	−4.6	−1.3	−7.8	−9.0	−11.4	−8.3
**III b**	3.8	11.6	12.4	12.3	10.9	9.9	11.7
**IV a 1**	−13.6	0.8	3.2	−2.6	−3.4	−6.4	−3.6
**IV a 2**	−11.4	2.6	4.5	−1.1	−1.3	−4.5	−1.6
**IV a 3**	−11.4	2.6	4.5	−1.1	−1.3	−4.5	−1.6
**IV a 4**	−13.6	0.8	3.2	−2.6	−3.4	−6.4	−3.6
**IV b**	−8.2	−5.8	−5.0	−6.3	−5.3	−6.9	−6.6
**MAE ^1^**		**11.7**	**13.8**	**8.9**	**8.2**	**5.6**	**8.3**

^1^ Mean average error with respect to SCS-MP2 averaged of all compounds.

## Supplementary Materials

The following are available online at https://www.mdpi.com/1420-3049/25/9/2064/s1: Syntheses and analytical data of the compounds; Crystallographic data for cpd. **1**; Mass spectrometry; Enzyme assays and hydrolysis assays; *T. b. brucei* cell survival assay; NMR spectra.
