# Supplementary material for: Naphthoquinones as Covalent Reversible Inhibitors of Cysteine Proteases—Studies on Inhibition Mechanism and Kinetics
Source: Molecules. 2020 Apr 28;25(9):2064. doi: 10.3390/molecules25092064 (PMC7248907; doi:10.3390/molecules25092064)
Supplement: Supplementary file 1 [file molecules-25-02064-s001.pdf]

molecules

Supplementary Material

Naphthoquinones as Covalent Reversible Inhibitors of Cysteine Proteases—Studies on Inhibition Mechanism and Kinetics

Philipp Klein [1], Fabian Barthels [2], Patrick Johe [2], Annika Wagner [3], Stefan Tenzer [4], Ute Distler [4], Thien Anh Le [5], Paul Schmid [5], Volker Engel [5], Bernd Engels [5], Ute A. Hellmich [3,6], Till Opatz [1,*] and Tanja Schirmeister [2,*]

[1] Department of Chemistry, Organic Chemistry Section, Johannes Gutenberg-Universität, Duesbergweg 10-14, 55128 Mainz, Germany; klein@uni-mainz.de (P.K.)
[2] Institute of Pharmaceutical and Biomedical Sciences, Johannes Gutenberg-Universität, Staudingerweg 5, 55128 Mainz, Germany; pajohe@uni-mainz.de (P.J.); barthels@uni-mainz.de (F.B.)
[3] Department of Chemistry, Biochemistry Section, Johannes Gutenberg-Universität, Johann-Joachim Becherweg 30, 55128 Mainz, Germany; a.wagner@uni-mainz.de (A.W.)
[4] Institute of Immunology, University Medical Center, Johannes Gutenberg-Universität Mainz, Langenbeckstr. 1, 55131 Mainz, Germany; tenzer@uni-mainz.de (S.T.); ute.distler@uni-mainz.de (U.D.)
[5] Institute of Physical and Theoretical Chemistry, Universität Würzburg, Emil-Fischer-Straße 42, 97074 Würzburg, Germany; thien_anh.le@physik.uni-wuerzburg.de (T.A.L.); paul.schmid@uni-wuerzburg.de (P.S.); volker.engel@uni-wuerzburg.de (V.E.); bernd.engels@uni-wuerzburg.de (B.E.)
[6] Centre for Biomolecular Magnetic Resonance (BMRZ), Goethe-University Frankfurt, Germany; u.hellmich@uni-mainz.de (U.A.H.)
* Correspondence: schirmei@uni-mainz.de (T.S.); Tel.: +49-(0)6131-39-25742; opatz@uni-mainz.de (T.O.); Tel.: +49-(0)6131-39-22272

1. Syntheses and analytical data of the compounds 2

2. Table 1: Crystalllographic data and structure refinement for Benzyl-*N*-(3-chloro-1,4-dioxo-1,4-dihydronaphthalen-2-yl)-L-phenylalanyl-L-leucinate (1) 14

3. Mass spectrometry 16

4. Enzyme assays and hydrolysis assays 17

5. *T. b. brucei* cell survival assay 19

6. NMR Spectra of the compounds 20

7. References 30

1. **Syntheses and analytical data of the compounds**

1.1. General Information

All reagents and solvents were obtained from commercial suppliers (Sigma Aldrich, Alfa Aesar, TCI chemicals, ABCR, Acros Organics and Fischer Scientific) and used without further purification. Flash column chromatography was performed using silica gel type 60 M (230-400 mesh, Macherey Nagel). Analytical thin-layer chromatography (TLC) was done on Merck silica gel plates (60 F_{254}) with defined solvent mixtures and visualized under UV light irradiation and/or TLC staining reagents. Melting points were determined in open capillary tube. IR spectra were measured with a JASCO (FT/IR-4100) with a diamond ATR unit and are reported in terms of frequency of absorption (ν, cm^{-1}). NMR experiments were performed on a 300 MHz (300 MHz ^{1}H and 75 MHz ^{13}C), a 400 MHz (400 MHz ^{1}H and 101 MHz ^{13}C) or a 600 MHz (600 MHz ^{1}H and 151 MHz ^{13}C) spectrometer from Bruker using deuterated solvents ((residual) solvent signals: $CDCl_3$: δH = 7.26 ppm, δC = 77.16 ppm; $(CD_3)_2SO$: δH = 2.50 ppm, δC = 39.52 ppm; CD_3OD: δH = 3.31 ppm, δC = 49.00 ppm) as internal references and reported in parts per million (ppm, δ) relative to tetramethylsilane (TMS, δ = 0.00 ppm)[1]. Coupling constants (*J*) are reported in Hz, and the multiplet abbreviations used were: s, singlet; d, doublet; t, triplet; m, multiplet; br, broad; and combinations thereof. Electrospray ionization (ESI-) mass spectra were recorded on an Agilent/Bruker LC/MSD trap XCT spectrometer or on a 1200-series HPLC-system (Agilent-Technologies) with binary pump, integrated diode array detector and Ascentis Express C18 column (2.7 µm, 30x2.1 mm). ESI-HRMS spectra were recorded on a Waters Q-TOF-Ultima 3 instrument or an Agilent 6545 QTOF-MS mass spectrometer. Preparative reverse phase separations were carried out on an Agilent 1290 Infinity II preparative system with a 1290 Infinity II preparative binary pump, 1260 Infinity II DAD and a 1290 Infinity II fraction collector. Optical rotations were measured with a Perkin–Elmer 241 polarimeter at 546 and 579 nm. Extrapolation to 589 nm was performed according to Thaler *et al.*[2].

1.2. Benzyl-N-(Tert-Butoxycarbonyl)-L-Phenylalanyl-L-Leucinate (A1)

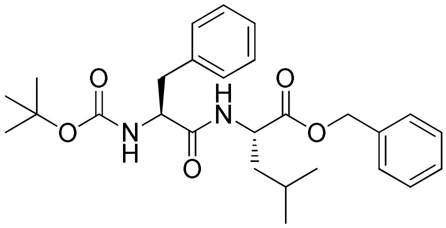

The product was synthesized according to Lawesson *et al.*[3]. In an inert gas atmosphere, *N*-Boc-L-phenylalanine (0.67 g, 2.54 mmol), L-leucine benzyl ester *p*-toluenesulfonate salt (1.00 g, 2.54 mmol) and triethylamine (360 µL, 2.54 mmol) were dissolved in dichloromethane (7 mL). The suspension was cooled to –18 °C and *N,N′*-dicyclohexylcarbodiimide (0.52 g, 2.54 mmol) was added. The resulting mixture was stirred for 1 h at that temperature and then 20 h at room temperature. The mixture was filtered off and the solvent was removed under reduced pressure. The residue was dissolved in ethyl acetate (10 mL) and washed with hydrochloric acid (0.1 M, 2.5 mL), sodium bicarbonate (0.5 M, 2.5 mL) and brine (2.5 mL). The organic phase was dried over magnesium sulfate and the solvent was removed under reduced pressure. The title compound was obtained as a colorless solid (970 mg, 2.07 mmol, 81 %) by crystallization from diethyl ether:petroleum ether (1:3 v/v, 4 mL).

$\mathbf{R_f}$=0.70 (petroleum ether:ethyl acetate, 2:1).

^{1}H-NMR, COSY (300 MHz, $CDCl_3$) δ=7.43–7.15 (m, 10H, H^{arom}), 6.28 (d, *J*=8.2 Hz, 1H, N*H*), 5.15 (d, *J*=12.3 Hz, 1H, OCH_2^{Bn}), 5.10 (d, *J*=12.3 Hz, 1H, OCH_2^{Bn}) , 5.00 (br s, 1H, N*H*), 4.61 (m, 1H), 4.34 (dd, *J*=7.3, 6.3 Hz, 1H), 3.05 (d, *J*=6.8 Hz, 2H, β-CH_2^{Phe}), 1.67–1.43 (m, 3H, γ-CH^{Leu}, β-CH_2^{Leu}), 1.42 (s, 9H, CH_3^{Boc}), 0.88 (d, *J*=6.1 Hz, 3H, δ-CH_3^{Leu}), 0.86 (d, *J*=6.1 Hz, 3H, δ-CH_3^{Leu}) ppm.

^{13}C-NMR, HMBC, HSQC (75 MHz, $CDCl_3$) δ=172.4 (*C*=O), 171.0 (*C*=O), 155.5 (*C*=O^{Boc}), 136.7 (γ-*C*^{Leu}), 135.5 (*C*$_q^{Bn}$), 129.5, 128.8, 128.7, 128.5, 128.4, 127.1 (*C*H^{arom}), 80.4 (*C*^{Boc,q}), 67.1 (*C*H_2^{Bn}), 55.8 (α-*C*H^{Phe}), 51.0 (α-*C*H^{Leu}), 41.7 (β-*C*H_2^{Leu}), 38.2 (β-*C*H_2^{Phe}), 28.4 (*C*H_3^{Boc}), 24.8 (γ-*C*H^{Leu}), 22.9, 22.0 (δ-*C*H_3^{Leu}) ppm.

ESI-MS (*m*/*z*): 469.3 (100) $[M+H]^+$, 491.3 (28) $[M+Na]^+$.

The spectroscopic data are in accordance with literature.[3]

1.3. Benzyl-L-Phenylalanyl-L-Leucinate Hydrochloride Salt (A2)

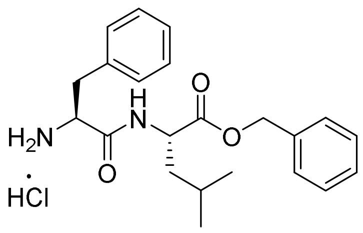

The deprotection was carried out similar to Hruby *et al.*[4]. In an inert gas atmosphere, the dipeptide **A1** (500 mg, 1.07 mmol) was added to HCl/ dioxane (4 M, 5 mL) at 0 °C. The ice bath was removed and the solution was stirred additional 30 min. The solvent was removed under reduced pressure, the product filtered off and washed with diethyl ether to produce a colorless solid (430 mg, 1.06 mmol, 99 %, Lit.[4]: <99 %) which was used for subsequent transformations without further purification.

^{1}H-NMR, COSY (300 MHz, DMSO-d_6) δ=9.03 (d, *J*=7.6 Hz, 1H, N*H*^{Leu}), 8.19 (br s, 3H, N*H*$_3^{Phe}$), 7.45–7.21 (m, 10H, *H*^{Ar}), 5.17 (d, *J*=12.4 Hz, 1H, OC*H*$_2^{Bn}$), 5.11 (d, *J*=12.4 Hz, 1H, OC*H*$_2^{Bn}$), 4.47–4.33 (m, 1H, α-C*H*^{Leu}), 4.07 (dd, *J*=8.0, 5.2 Hz, 1H, α-C*H*^{Phe}), 3.11 (dd, *J*=14.1, 5.2 Hz, 1H, β-C*H*$_2^{Phe}$), 2.89 (dd, *J*=14.1, 8.1 Hz, 1H, β-C*H*$_2^{Phe}$), 1.61 (m, 3H, β-C*H*$_2^{Leu}$, γ-C*H*^{Leu}), 0.90 (d, *J*=6.4 Hz, 3H, δ-C*H*$_3^{Leu}$), 0.86 (d, *J*=6.3 Hz, 3H, δ-C*H*$_3^{Leu}$) ppm.

The spectroscopic data are in accordance with literature.[4]

1.4. Benzyl-L-Phenylalanyl-L-Leucinate Trifluoroacetic Acid Salt (A10)

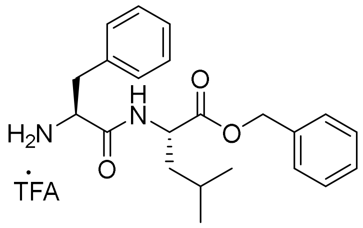

The product was obtained by deprotection with trifluoroacetic acid. The dipeptide **A1** (113 mg, 0.24 mmol) was added to a mixture of TFA (1 mL) and dichloromethane (2 mL) at 0 °C. After 10 min. the ice bath was removed and the solution was stirred for 2 h at room temperature. The solvent was removed under reduced pressure and the product (116 mg, 0.24 mmol, >99 %) was obtained as a colorless solid which was used without further purification.

^{1}H-NMR (300 MHz, DMSO-d_6) δ=8.89 (d, *J*=7.7 Hz, 1H, N*H*^{Leu}), 8.11 (br s, 3H, N*H*$_3^{Phe}$), 7.37 – 7.13 (m, 10H, *H*^{Ar}), 5.17 (d, *J*=12.4 Hz, 1H, OC*H*$_2^{Bn}$), 5.11 (d, *J*=12.4 Hz, 1H, OC*H*$_2^{Bn}$), 4.41 (q, *J*=7.5 Hz, 1H), 4.03 (dd, *J*=8.3, 5.0 Hz, 1H), 3.08 (dd, *J*=14.2, 4.9 Hz, 1H, β-C*H*$_2^{Phe}$), 2.86 (dd, *J*=14.2, 8.4 Hz, 1H, β-C*H*$_2^{Phe}$), 1.70 – 1.53 (m, 3H, β-C*H*$_2^{Leu}$, γ-C*H*^{Leu}), 0.91 (d, *J*=6.2 Hz, 3H, δ-C*H*$_3^{Leu}$), 0.86 (d, *J*=6.2 Hz, 3H, δ-C*H*$_3^{Leu}$) ppm.

1.5. N-(tert-Butoxycarbonyl)-L-Phenylalanyl-L-Leucine (A3)

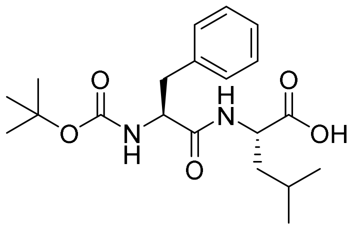

Benzyl *N*-(*tert*-butoxycarbonyl)-L-phenylalanyl-L-leucinate (120 mg, 0.26 mmol) was dissolved in THF (5 mL). Pd/C (20 mg) was added and the mixture was stirred under a hydrogen atmosphere for 2 h. The suspension was filtered over a pad of celite and washed with THF (15 mL). The solvent was evaporated under reduced pressure and the remaining colorless oil (89 mg, 0.26 mmol) was used without further purification.

ESI-MS: m/z = 2.9 min., 279.1 (100%, $[M - Boc]^+$, calc. 279.2).

1.6. L-Phenylalanyl-L-Leucine Trifluoroacetic Acid Salt (A4)

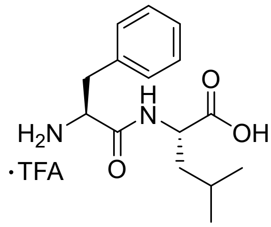

Crude *N*-(*tert*-butoxycarbonyl)- L-phenylalanyl-L-leucine (89 mg, 0.26 mmol) was dissolved in dichloromethane (2 mL) and TFA (1 mL) was added dropwise at room temperature. After stirring for 1 h, the solvents were removed in a stream of nitrogen. Remaining solvent was removed by co-evaporation with chloroform, toluene and ethanol (5 mL each) to yield a colorless solid (88 mg, 0.22 mmol).

ESI-MS: m/z = 0.7 min., 279.1 (100%, $[M+H]^+$, calc. 279.2).

1.7. Methyl-N-(tert-butoxycarbonyl)-L-phenylalanyl-L-leucinate (A5)

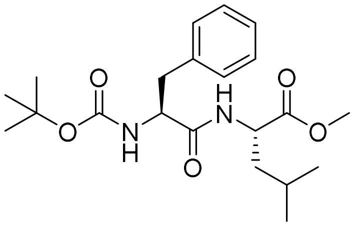

A solution of leucine methyl ester hydrochloride (500 mg, 2.75 mmol), Boc-phenylalanine (730 mg, 2.75 mmol) and triethylamine (380 µL, 2.75 mmol) in dichloromethane (4 mL) was cooled to -18 °C in an ice salt bath. DCC (567 mg, 2.75 mmol) in dichloromethane (1.5 + 1.0 mL) was added slowly and the mixture was left to warm to room temperature overnight. After 16 h, the suspension was filtered, washed with dichloromethane and the solvent evaporated. The residue was dissolved in ethyl acetate (150 mL) and washed with 0.1 N HCl, 0.5 N NaHCO3 and brine (50 mL each). Pure product (833 mg, 2.12 mmol, 77 %) was obtained as a colorless solid by chromatography (SiO_2, cyclohexane:ethyl acetate (9:1 to 1:9)).

mp: 105–106 °C.

^{1}H-NMR (300 MHz, $CDCl_3$) δ=7.35–7.16 (m, 5H, H^{Ar}), 6.24 (d, *J*=8.3 Hz, 1H, N*H*), 4.99 (br s, 1H, N*H*), 4.57 (td, *J*=8.5, 5.2 Hz, 1H, α-C*H*), 4.34 (q, *J*=7.1 Hz, 1H, α-C*H*), 3.69 (s, 3H, OMe), 3.07 (d, *J*=6.8 Hz, 2H, CH_2^{Phe}), 1.66–1.45 (m, 3H, β-CH_2^{Leu}, γ-CH^{Leu}), 1.41 (s, 9H, ^{t}Bu), 0.90 (d, *J*=6.0 Hz, 3H, δ-CH_3^{Leu}), 0.88 (d, *J*=6.1 Hz, 3H, δ-CH_3^{Leu}) ppm.

ESI-MS (*m*/*z*): 293.3 (100) [M - Boc+H]$^+$, 415.3 (69) [M+Na]$^+$.

The spectroscopic data are in accordance with literature.[16]

1.8. Methyl-L-phenylalanyl-L-leucinate trifluoroacetic acid salt (A6)

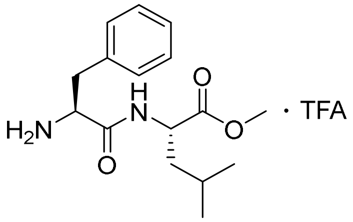

Methyl-*N*-(*tert*-butoxycarbonyl)-L-phenylalanyl-L-leucinate (221 mg, 0.56 mmol) was dissolved in dichloromethane (2 mL) and cooled to 0 °C. TFA (2 mL) in DCM (2 mL) was added dropwise. After stirring for 3 h, the solvents were removed in a stream of nitrogen. Remaining solvent was removed by co-evaporation with chloroform, toluene and ethanol (5 mL each) to yield a colorless solid. The product was used without further purification

ESI-MS (*m*/*z*): 293.3 (100) [M+H]$^+$, 315.2 (15) [M+Na]$^+$.

1.9. tert-Butyl-N-((benzyloxy)carbonyl)-L-phenylalanyl-L-leucinate (A7)

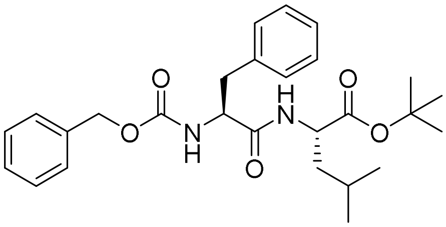

A solution of Z-protected phenylalanine (302 mg, 1.01 mmol), leucine *tert*-butyl ester (1.00 mmol), HOBt monohydrate (154 mg, 1.01 mmol), EDC hydrochloride (194 mg, 1.01 mmol) and DMAP (14 mg, 0.11 mmol) in DCM (5 mL) was cooled to 0 °C. Triethylamine (139 µL, 1.00 mmol) was slowly added and the mixture was left to warm to room temperature. After 2 d, DCM (10 mL) was added and the organic phase was washed with sat. NH_4Cl, water and brine (15 mL each). Pure product (356 mg, 0.76 mmol, 76 %) was obtained as a colorless solid by chromatography (SiO_2, cyclohexane:ethyl acetate (9:1 to 1:9)).

$\mathbf{R_f}$=0.22 (cyclohexane:ethyl acetate, 5:1).

mp: 89–91 °C.

^{1}H-NMR, COSY (300 MHz, $CDCl_3$) δ=7.41–7.13 (m, 10H, H^{Ar}), 6.31 (d, *J*=8.2 Hz, 1H, NH^{Leu}), 5.38 (d, *J*=8.3 Hz, 1H, NH^{Phe}), 5.08 (s, 2H, OCH_2^{Bn}), 4.51–4.37 (m, 2H, α-C$H^{Phe,Leu}$), 3.08 (d, *J*=6.8 Hz, 2H, β-CH_2^{Phe}), 1.60–1.41 (m, 3H, β-CH_2^{Leu}, γ-CH^{Leu}), 1.45 (s, 9H, ^{t}Bu), 0.90 (d, *J*=6.0 Hz, 3H, δ-CH_3^{Leu}), 0.89 (d, *J*=6.2 Hz, 3H, δ-CH_3^{Leu}) ppm.

^{13}C-NMR, HMBC, HSQC (75 MHz, $CDCl_3$) δ=171.7 (*C*=O^{Leu}), 170.4 (*C*=O^{Phe}), 156.0 (*C*OO), 136.3, 136.3, 129.5, 128.7, 128.6, 128.3, 128.1, 127.1 (C^{arom}), 82.0 (C^tBu), 67.1 (CH_2^{Bn}), 56.1 (α-CH^{Phe}), 51.5 (α-CH^{Leu}), 41.9 (β-CH_2^{Leu}), 38.5 (β-CH_2^{Phe}), 28.1 (^{t}Bu), 24.9 (γ-CH^{Leu}), 22.8, 22.2 (δ-CH_3^{Leu}) ppm.

ESI-MS (*m*/*z*): 413.4 (74) [M - O^{t}Bu+H_2O]$^+$, 491.3 (100) [M+Na]$^+$.

ESI-HRMS (*m*/*z*) calculated for $[C_{27}H_{36}N_2NaO_5]^+$= 491.2516, found 491.2512.

The spectroscopic data are in accordance with literature.[17]

1.10. tert-Butyl-L-phenylalanyl-L-leucinate (A8)

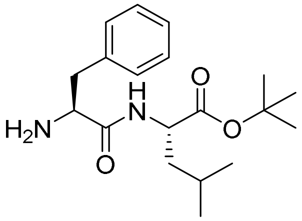

To a solution of *tert*-butyl-*N*-((benzyloxy)carbonyl)-L-phenylalanyl-L-leucinate (325 mg, 0.694 mmol) in THF (30 mL) was added palladium on activated charcoal (5 wt%, 20 mg). The reaction flask was evacuated and vented with hydrogen three times. After 19 h of stirring at room temperature, another portion of Pd/C (5 wt%, 20 mg) was added. After a total of 43 h, the mixture was filtered through celite to obtain the product (193 mg, 0.58 mmol, 84 %) as a colorless oil, which was used without further purification.

^{1}H-NMR (300 MHz, $CDCl_3$) δ=7.59 (d, *J*=7.9 Hz, 1H, NH^{Leu}), 7.35–7.16 (m, 5H, H^{Ar}), 4.44–4.31 (m, 1H, α-C*H*), 4.19–4.11 (m, 1H, α-C*H*), 3.28 (dd, *J*=13.9, 5.7 Hz, 1H, β-CH_2^{Phe}), 3.09 (dd, *J*=13.7, 6.8 Hz, 1H, β-CH_2^{Phe}), 1.70–1.54 (m, 3H, γ-CH^{Leu}, β-CH_2^{Leu}), 1.43 (s, 9H, ^{t}Bu), 0.89 (d, *J*=6.2 Hz, 3H, δ-CH_3^{Leu}), 0.88 (d, *J*=6.2 Hz, 3H, δ-CH_3^{Leu}) ppm.

ESI-MS (*m*/*z*): 279.2 (100) [M - O^{t}Bu+H_2O]$^+$, 335.2 (13) [M+H]$^+$, 357.2 (7) [M+Na]$^+$.

The spectroscopic data are in accordance with literature.[18]

1.11. 2,3-Dicyano-1,4-naphthoquinone (A9)

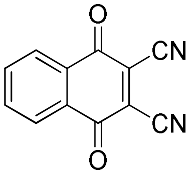

The product was synthesized according to Budni *et al.*[6]. A solution of sodium cyanide (1.33 g, 20.5 mmol) in water (6 mL) was added to a solution of 2,3-dichloro-1,4-naphthoquinone (1.00 g, 4.4 mmol) in ethanol (17 mL) in a rate that the temperature did not exceed 40 °C. The mixture was stirred at room temperature for 3 h and then acidified with cold, concentrated hydrochloric acid to pH=1. The resulting solid was filtered, washed with water and dried under reduced pressure. The crude product was suspended in acetic acid (10 mL) and heated to 100 °C. While heating, 30 % nitric acid (1.5 mL) was added. Addition of ice to the cold solution and collection of the precipitate afforded the desired product (319 mg, 1.53 mmol, 35 %) as a yellow solid.

R_f=0.60 (petroleum ether:ethyl acetate, 5:3).

mp: >200 °C.

^{1}H-NMR (400 MHz, $CDCl_3$) δ=8.27–8.18 (m, 2H), 8.00–7.92 (m, 2H) ppm.

^{13}C-NMR (101 MHz, $CDCl_3$) δ=176.2, 136.4, 130.4, 129.8, 128.3, 110.4 ppm.

IR: ν=1669, 1604, 1584, 1457, 1332, 1293, 1262, 1188, 979, 899, 863, 791, 711 cm^{-1}.

ESI-HRMS (*m*/*z*) calculated for $[C_{12}H_4N_2O_2Na]^+$=231.0170, found 231.0169.

1.12. Benzyl-N-(3-chloro-1,4-dioxo-1,4-dihydronaphthalen-2-yl)-L-phenylalanyl-L-leucinate (1)

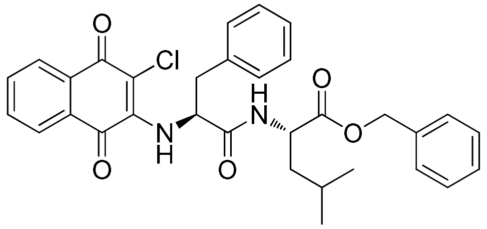

To a solution of 2,3-dichloro-1,4-naphthoquinone (114 mg, 0.50 mmol) and the hydrochloride of the deprotected dipeptide **A2** (202 mg, 0.50 mmol) in dichloromethane (4 mL) was slowly added a solution of triethylamine (70 µL, 0.50 mmol) in dichloromethane (1 mL). After 24 h at room temperature, the solution was diluted with dichloromethane and washed with water, 0.5 M sodium bicarbonate solution and brine. Purification by chromatography (SiO_2, petroleum ether:ethyl acetate (4:1)) afforded the title compound (179 mg, 0.32 mmol, 64 %) as a red solid. Crystallization from ethanol afforded crystals suitable for X-ray crystallography (see **Table 1**).

R$_f$=0.30 (petroleum ether:ethyl acetate, 4:1).

mp: 129–132 °C.

^{1}H-NMR, COSY (300 MHz, $CDCl_3$) δ=8.11 (dd, *J*=7.6, 1.4 Hz, 1H, *H*-5/8^{Naph}), 7.99 (dd, *J*=7.6, 1.4 Hz, 1H, *H*-5/8^{Naph}), 7.71 (td, *J*=7.5, 1.4 Hz, 1H, *H*-6/7^{Naph}), 7.63 (td, *J*=7.5, 1.4 Hz, 1H, *H*-6/7^{Naph}), 7.39–7.16 (m, 10H, *H*^{Ar}), 6.40 (d, *J*=8.0 Hz, 1H, N*H*^{Phe}), 6.11 (d, *J*=8.1 Hz, 1H, N*H*^{Leu}), 5.25 (dt, *J*=8.1, 6.6 Hz, 1H, α-C*H*^{Phe}), 5.14 (d, *J*=12.2 Hz, 1H, OC*H*$_2$^{Bn}), 5.08 (d, *J*=12.2 Hz, 1H, OC*H*$_2$^{Bn}), 4.70–4.60 (m, 1H, α-C*H*^{Leu}), 3.22 (dd, *J*=13.8, 6.5 Hz, 1H, β-C*H*$_2$^{Phe}), 3.14 (dd, *J*=13.8, 6.6 Hz, 1H, β-C*H*$_2$^{Phe}), 1.64–1.42 (m, 3H, β-C*H*$_2$, γ-C*H*^{Leu}), 0.88 (d, *J*=6.1 Hz, 3H, δ-C*H*$_3$^{Leu}), 0.87 (d, *J*=6.3 Hz, 3H, δ-C*H*$_3$^{Leu}) ppm.

^{13}C-NMR, HMBC, HSQC (75 MHz, $CDCl_3$) δ=180.2, 176.8 (*C*-1/4^{Naph}), 172.1 (*C*=O^{Leu}), 170.4 (*C*=O^{Phe}), 143.9 (*C*-3^{Naph}), 135.5 (γ-*C*^{Phe}), 135.3 (*C*$_q$^{Bn}), 135.0 (*C*-6/7^{Naph}), 132.9 (*C*-6/7^{Naph}), 132.2, 130.0, 129.6, 129.0, 128.7, 128.6, 128.4, 127.6 (*C*H^{Ar}), 127.0 (*C*-5/8^{Naph}), 126.9 (*C*-5/8^{Naph}), 67.3 (*C*H$_2$^{Bn}), 58.9 (α-*C*H^{Phe}), 51.3 (α-*C*H^{Leu}), 41.7 (β-*C*H$_2$^{Leu}), 40.6 (β-*C*H$_2$^{Phe}), 24.9 (γ-*C*H^{Leu}), 22.8 (δ-*C*H$_3$^{Leu}), 22.1 (δ-*C*H$_3$^{Leu}) ppm.

IR: ν=3309, 2957, 1739, 1674, 1594, 1568, 1455, 1386, 1330, 1291, 1265, 1146, 990, 910, 844, 789, 720, 697, 682 cm^{-1}.

ESI-HRMS (*m/z*) calculated for $[C_{32}H_{31}N_2O_5Na^{35}Cl]^+$=581.1819, found 581.1802.

$[\boldsymbol{\alpha}]_D^{24}$= –28.0° (c=1.0, CH_2Cl_2).

1.13. Benzyl-N-(3-cyano-1,4-dioxo-1,4-dihydronaphthalen-2-yl)-L-phenylalanyl-L-leucinate (2)

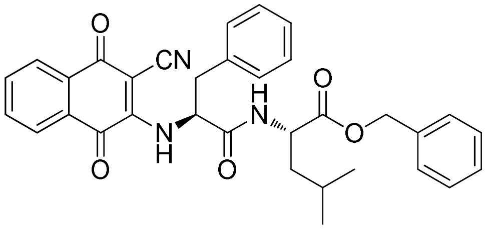

To a suspension of the hydrochloride of dipeptide **A2** (145 mg, 0.30 mmol) in dichloromethane was slowly added triethylamine (41 µL, 0.30 mmol). After 10 min. of stirring, naphthoquinone **A9** (62 mg, 0.30 mmol) was added and the resulting solution was stirred for 4 h at room temperature. After evaporation of the solvent, pure product (126 mg, 0.23 mmol, 76 %) was obtained by chromatography (SiO_2, petroleum ether:ethyl acetate (5:1)).

R$_f$=0.28 (petroleum ether:ethyl acetate, 3:1).

mp: 67–70 °C.

^{1}H-NMR, COSY (300 MHz, $CDCl_3$) δ=8.16 (dd, *J*=7.8, 1.2 Hz, 1H), 8.11 (dd, *J*=7.8, 1.3 Hz, 1H, H-5/8^{Naph}), 7.81 (m, 2H, *H*-6/7^{Naph}, N*H*^{Phe}), 7.70 (td, *J*=7.5, 1.4 Hz, 1H, *H*-6/7^{Naph}), 7.41–7.32 (m, 5H, *H*^{Ar}), 7.28–7.20 (m, 5H, *H*^{Ar}), 6.13 (d, *J*=7.8 Hz, 1H, N*H*^{Leu}), 5.34 (ddd, *J*=8.9, 7.4, 6.3 Hz, 1H, α-C*H*^{Phe}), 5.18 (d, *J*=12.2 Hz, 1H, OC*H*$_2$^{Bn}), 5.11 (d, *J*=12.2 Hz, 1H, OC*H*$_2$^{Bn}), 4.62–4.53 (m, 1H, α-C*H*^{Leu}), 3.35 (dd, *J*=13.8, 6.2 Hz, 1H, β-C*H*$_2$^{Phe}), 3.16 (dd, *J*=13.8, 7.5 Hz, 1H, β-C*H*$_2$^{Phe}), 1.66–1.49 (m, 3H, β-C*H*$_2$^{Leu}, γ-C*H*^{Leu}), 0.88 (d, *J*=5.3 Hz, 6H, δ-C*H*$_3$^{Leu}) ppm.

^{13}C-NMR, HMBC, HSQC (75 MHz, $CDCl_3$) δ=179.1, 178.5 (*C*-1/4^{Naph}), 171.7 (*C*=O^{Leu}), 168.3 (*C*=O^{Phe}), 148.4, 136.0 (*C*-6/7^{Naph}), 135.3 (*C*$_q$^{Bn}), 134.5 (*C*$_q$^{Phe}), 133.5 (*C*-6/7^{Naph}), 132.6, 129.7 (*C*-4a/8a^{Naph}), 129.7, 129.1, 128.8, 128.7, 128.5, 127.8, 127.6, 127.2 (*C*-5/8^{Naph}), 86.4 (*C*-3^{Naph}), 67.3 (*C*H$_2$^{Bn}), 56.7 (α-*C*H^{Phe}), 51.6 (α-*C*H^{Leu}), 41.4 (β-*C*H$_2$^{Leu}), 40.0 (β-*C*H$_2$^{Phe}), 24.9 (γ-*C*H^{Leu}), 22.7 (δ-*C*H$_3$^{Leu}), 22.1 (δ-*C*H$_3$^{Leu}) ppm.

IR: ν=3287, 2957, 2212, 1740, 1685, 1599, 1576, 1525, 1455, 1387, 1332, 1288, 1187, 1149, 1081, 966, 895, 812, 793, 749, 724, 697, 667 cm^{-1}.

ESI-HRMS (*m*/*z*) calculated for $[C_{33}H_{32}N_3O_5]^+$=550.2342, found 550.2324.

$[\boldsymbol{\alpha}]_{D}^{24}$= –55.3° (c=1.0, CH_2Cl_2).

1.14. (3-Chloro-1,4-dioxo-1,4-dihydronaphthalen-2-yl)-L-phenylalanyl-L-leucine (3)

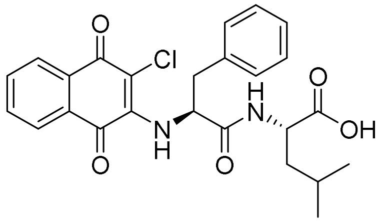

To the crude mixture of the deprotected dipeptide **A4** (54 mg, 137 µmol) were added triethylamine (29 µL, 210 µmol), 2,3-dichloro-1,4-naphthoquinone (34 mg, 150 µmol) and ethanol (1.5 mL). The mixture was stirred at 60 °C for 23 h. More triethylamine (29 µL, 210 µmol) was added and the mixture was stirred at 65 °C for another 14 h. The solvent was removed and the residue taken up in ethyl acetate (15 mL). The organic phase was washed with water, 1 N HCl and brine (10 mL each). Chromatography (SiO_2, dichloromethane: methanol (15:1)) afforded product (16 mg, 34 µmol, 25 %) as a red amorphous solid.

R$_f$=0.25 (chloroform:methanol, 10:1).

^{1}H-NMR, COSY (600 MHz, CD_3OD) δ=7.99 (d, *J*=7.6 Hz, 1H, *H*-5/8^{Naph}), 7.94 (br d, *J*=7.6 Hz, 1H, *H*-5/8^{Naph}), 7.74 (td, *J*=7.6, 1.3 Hz, 1H, *H*-6/7^{Naph}), 7.67 (td, *J*=7.6, 1.3 Hz, 1H, *H*-6/7^{Naph}), 7.28 (m, 2H, *H*^{Ar}), 7.21 (t, *J*=7.4 Hz, 2H, *H*^{Ar}), 7.14 (t, *J*=7.4 Hz, 1H, *H*^{Ar}), 5.46 (dd, *J*=8.0, 4.7 Hz, 1H, α-C*H*^{Phe}), 4.44 (t, *J*=7.4 Hz, 1H, α-C*H*^{Leu}), 3.35 (dd, *J*=14.1, 4.6 Hz, 1H, β-C*H*$_2$^{Phe}), 3.10 (dd, *J*=14.0, 8.0 Hz, 1H, β-C*H*$_2$^{Phe}), 1.79–1.72 (m, 1H, γ-C*H*^{Leu}), 1.69–1.66 (m, 2H, β-C*H*$_2$^{Leu}), 0.97 (d, *J*=6.6 Hz, 3H, δ-C*H*$_3$^{Leu}), 0.93 (d, *J*=6.5 Hz, 3H, δ-C*H*$_3$^{Leu}) ppm.

^{13}C-NMR, HMBC, HSQC (151 MHz, CD_3OD) δ=180.9, 178.2 (*C*-1/4^{Naph}), 173.1 (*C*=O^{Phe}), 137.3 (*C*$_q$^{Ar}), 135.9, 134.0 (*C*-6/7^{Naph}), 133.4, 131.4 (*C*-4a/8a^{Naph}), 130.9, 129.6, 128.1 (*C*^{Ar}), 127.8, 127.3 (*C*-5/8^{Naph}), 59.4 (α-*C*H^{Phe}), 53.5 (α-*C*H^{Leu}), 42.4 (β-*C*H$_2$^{Leu}), 41.3 (β-*C*H$_2$^{Phe}), 26.1 (γ-*C*H^{Leu}), 23.6 (δ-*C*H$_3$^{Leu}), 21.9 (δ-*C*H$_3$^{Leu}) ppm.

*acid carbonyl, *C*-2^{Naph} and *C*-3^{Naph} not observed

IR: ν=3307, 2957, 1717, 1653, 1601, 1332, 1293, 1266, 721 cm^{-1}.

ESI-MS (*m/z*): 469.4 (100) $[M+H]^+$, 491.2 (9) $[M+Na]^+$.

ESI-HRMS (*m/z*) calculated for $[C_{25}H_{26}{}^{35}ClN_2O_5]^+$= 469.1525, found 469.1522; calculated for $[C_{25}H_{26}{}^{37}ClN_2O_5]^+$= 471.1496, found 471.1503.

$[\boldsymbol{\alpha}]_D^{24}$= -9.4° (c=0.16, $CHCl_3$).

1.15. (3-Cyano-1,4-dioxo-1,4-dihydronaphthalen-2-yl)-L-phenylalanyl-L-leucine (4)

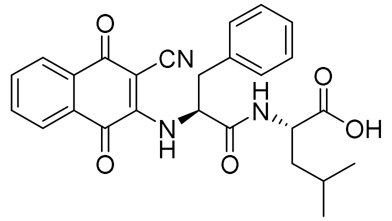

To the crude mixture of the deprotected dipeptide **A4** (55 mg, 140 μmol) were added triethylamine (29 μL, 210 μmol), corresponding naphthoquinone **A9** (30 mg, 144 μmol) and ethanol (1.5 mL). The mixture was stirred at 60 °C for 23 h. More triethylamine (29 μL, 210 μmol) was added and the mixture was stirred at 65 °C for another 14 h. The solvent was removed and the residue taken up in ethyl acetate (15 mL). The organic phase was washed with water, 1N HCl and brine (10 mL each). Chromatography (SiO_2, dichloromethane: methanol (15:1)) afforded product (30 mg, 65 μmol, 46 %) which was further purified by preparative HPLC (ACE C_{18}PFP, 5 μm, 150 mm × 30 mm, 37.5 mL/min, MeCN:water 1:1).

R$_f$=0.14 (dichloromethane:methanol, 15:1).

^{1}H-NMR, COSY (600 MHz, $CDCl_3$) δ=8.19 (m, 2H, *H*-5/8^{Naph}, N*H*^{Phe}), 8.15 (dd, *J*=7.7, 1.3 Hz, 1H, *H*-5/8^{Naph}), 7.84 (m, 2H, *H*-6/7^{Naph}, N*H*^{Leu}), 7.73 (td, *J*=7.5, 1.3 Hz, 1H, *H*-6/7^{Naph}), 7.20 (t, *J*=7.5, 2H, *H*^{Ar}), 7.13 (d, *J*=7.4, 1H, *H*^{Ar}), 7.10 (m, 2H, *H*^{Ar}), 5.59 (dt, *J*=9.3, 6.3 Hz, 1H, *α*-C*H*^{Phe}), 4.75 (m, 1H, *α*-C*H*^{Leu}), 3.24 (dd, *J*=13.8, 6.1 Hz, 1H, β-C*H*$_2$^{Phe}), 3.19 (dd, *J*=13.8, 6.5 Hz, 1H, β-C*H*$_2$^{Phe}), 1.74–1.67 (m, 2H, β-C*H*$_2$^{Leu}), 1.65–1.58 (m, 1H, γ-C*H*^{Leu}), 0.96 (d, *J*=5.9 Hz, 6H, δ-C*H*$_3$^{Leu}) ppm.

^{13}C-NMR, HMBC, HSQC (151 MHz, $CDCl_3$) δ=179.1, 178.0 (*C*-1/4^{Naph}), 175.7 (*C*=O^{Leu}), 168.1 (*C*=O^{Phe}), 148.7 (*C*-3^{Naph}), 136.2 (*C*-6/7^{Naph}), 134.2 (*C$_q$*^{Ar}), 133.6 (*C*-6/7^{Naph}), 132.6 (*C*-4a/8a^{Naph}), 130.0 (*C*^{Ar}), 129.7 (*C*-4a/8a^{Naph}), 128.7, 127.9 (*C*^{Ar}), 127.9, 127.2 (*C*-5/8^{Naph}), 115.4 (-*C*N), 84.5 (*C*-2^{Naph}), 55.9 (*α*-CH^{Phe}), 51.3 (*α*-*C*H^{Leu}), 43.0 (β-*C*H$_2$^{Leu}), 40.8 (β-*C*H$_2$^{Phe}), 24.9 (γ-*C*H^{Leu}), 22.7 (δ-*C*H$_3$^{Leu}), 22.6 (δ-*C*H$_3$^{Leu}) ppm.

IR: ν=3313, 2958, 2212, 1684, 1599, 1575, 1559, 1540, 1286, 726 cm^{-1}.

ESI-MS (*m/z*): 460.4 (36) $[M+H]^+$, 482.3 (100) $[M+Na]^+$.

ESI-HRMS (*m/z*) calculated for $[C_{26}H_{25}N_3NaO_5]^+$= 482.1686, found 482.1680.

$[\boldsymbol{\alpha}]_D^{23}$= +30.6° (c=0.16, $CHCl_3$).

1.16. Methyl-N-(3-chloro-1,4-dioxo-1,4-dihydronaphthalen-2-yl)-L-phenylalanyl-L-leucinate (5)

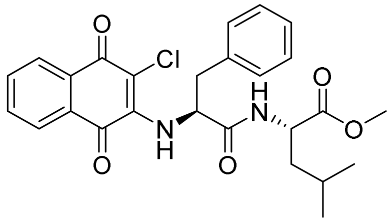

To a crude mixture of the deprotected dipeptide **A6** (76 mg, 188 μmol) in ethanol (1 mL) were added triethylamine (40 μL, 282 μmol) and 2,3-dichloro-1,4-naphthoquinone (49 mg, 216 μmol). After

14 h at 80 °C, more triethylamine (40 µL, 282 µmol) was added and the mixture stirred for an additional 22 h. After cooling to room temperature, ethyl acetate (15 mL) was added and the organic phase was washed 3 times with water and with brine (10 mL each). The organic phase was dried over sodium sulfate. Pure product (64 mg, 133 µmol, 71 %) was obtained as a red amorphous solid by chromatography (SiO_2, cyclohexane:ethyl acetate (9:1 to 1:9)).

R$_f$=0.11 (cyclohexane:ethyl acetate, 5:1).

^{1}H-NMR, COSY (400 MHz, $CDCl_3$) δ=8.11 (dd, *J*=7.7, 1.3 Hz, 1H), 8.01 (dd, *J*=7.6, 1.4 Hz, 1H, *H*-5/8^{Naph}), 7.71 (td, *J*=7.5, 1.4 Hz, 1H), 7.63 (td, *J*=7.6, 1.4 Hz, 1H, *H*-6/7^{Naph}), 7.33–7.21 (m, 5H, *H*^{Ar}), 6.43 (br d, *J*=8.1 Hz, 1H, N*H*^{Phe}), 6.04 (d, *J*=8.2 Hz, 1H, N*H*^{Leu}), 5.25 (dt, *J*=8.1, 6.6 Hz, 1H, α-C*H*^{Phe}), 4.60 (td, *J*=8.4, 5.3 Hz, 1H, α-C*H*^{Leu}), 3.68 (s, 3H, OMe), 3.24 (dd, *J*=13.8, 6.5 Hz, 1H), 3.16 (dd, *J*=13.8, 6.8 Hz, 1H, β-C*H*$_2$^{Phe}), 1.62–1.42 (m, 3H, β-C*H*$_2$^{Leu}, γ-C*H*^{Leu}), 0.90 (d, *J*=6.5 Hz, 3H, δ-C*H*$_3$^{Leu}), 0.88 (d, *J*=6.5 Hz, 3H, δ-C*H*$_3$^{Leu}) ppm.

^{13}C-NMR, HMBC, HSQC (101 MHz, $CDCl_3$) δ=180.2, 176.8 (*C*-1/4^{Naph}), 172.7 (*C*=O^{Leu}), 170.4 (*C*=O^{Phe}), 143.9 (*C*-3^{Naph}), 135.6 (*C*$_q$^{Ar}), 135.0, 132.9 (*C*-6/7^{Naph}), 132.3, 130.1 (*C*-4a/8a^{Naph}), 129.7, 129.0, 127.6 (*C*^{Ar}), 127.0, 126.9 (*C*-5/8^{Naph}), 110.1 (*C*-Cl), 58.9 (α-*C*H^{Phe}), 52.5 (OMe), 51.1 (α-*C*H^{Leu}), 41.7 (β-*C*H$_2$^{Leu}), 40.6 (β-*C*H$_2$^{Phe}), 24.9 (γ-*C*H^{Leu}), 22.8 (δ-*C*H$_3$^{Leu}), 22.1 (δ-*C*H$_3$^{Leu}) ppm.

IR: ν=3312, 3066, 3031, 2957, 2871, 1743, 1677, 1602, 1572, 1521, 1455, 1439, 1332, 1293, 1267, 1207 cm^{-1}.

ESI-MS (*m/z*): 483.4 (100) $[M+H]^+$, 505.3 (40) $[M+Na]^+$.

ESI-HRMS (*m/z*) calculated for $[C_{26}H_{28}{}^{35}ClN_2O_5]^+$= 483.1681, found 483.1682; calculated for $[C_{26}H_{28}{}^{37}ClN_2O_5]^+$= 485.1652, found 483.1664.

$[\boldsymbol{\alpha}]_D^{22}$= –36.3° (c=0.65, $CHCl_3$).

1.17. Methyl-N-(3-cyano-1,4-dioxo-1,4-dihydronaphthalen-2-yl)-L-phenylalanyl-L-leucinate (6)

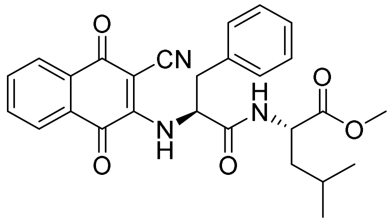

To the crude mixture of the deprotected dipeptide **A6** (30 mg, 74 mmol) was added triethylamine (15 µL, 110 µmol), the corresponding naphthoquinone **A9** (23 mg, 110 µmol) and ethanol (2 mL). The mixture was stirred at 80 °C for 17.5 h. After cooling to room temperature, ethyl acetate was added (50 mL) and the organic phase was washed with water and 2 times brine (50 mL each). The organic phase was dried over sodium sulfate. Pure product (29 mg, 61 µmol, 55 %) was obtained as a yellow oil by chromatography (SiO_2, cyclohexane:ethyl acetate (9:1 to 1:9)).

R$_f$=0.31 (cyclohexane:ethyl acetate, 2:1).

^{1}H-NMR, COSY (400 MHz, $CDCl_3$) δ=8.15 (dd, *J*=7.0, 1.3 Hz, 1H, *H*-5/8^{Naph}), 8.10 (dd, *J*=7.8, 1.3 Hz, 1H, *H*-5/8^{Naph}), 7.81 (m, 2H, *H*-6/7^{Naph}, N*H*^{Phe}), 7.70 (td, *J*=7.6, 1.3 Hz, 1H, *H*-6/7^{Naph}), 7.32–7.21 (m, 5H, *H*^{Ar}), 6.24 (d, *J*=7.8 Hz, 1H, N*H*^{Leu}), 5.35 (ddd, *J*=8.9, 7.6, 6.2 Hz, 1H, α-C*H*^{Phe}), 4.54 (td, *J*=8.1, 5.4 Hz, 1H, α-C*H*^{Leu}), 3.70 (s, 3H, OMe), 3.39 (dd, *J*=13.8, 6.2 Hz, 1H, β-C*H*$_2$^{Phe}), 3.21 (dd, *J*=13.8, 7. 6 Hz, 1H, β-C*H*$_2$^{Phe}), 1.68–1.48 (m, 3H, β-C*H*$_2$^{Leu}, γ-C*H*^{Leu}), 0.90 (d, *J*=6.0 Hz, 6H, δ-C*H*$_3$^{Leu}) ppm.

^{13}C-NMR, HMBC, HSQC (101 MHz, $CDCl_3$) δ=179.1, 178.5 (*C*-1/4^{Naph}), 172.3 (*C*=O^{Leu}), 168.4 (*C*=O^{Phe}), 148.4 (*C*-4a/8a^{Naph}), 136.0 (*C*-6/7^{Naph}), 134.6 (*C*^{Ar}), 133.5 (*C*-6/7^{Naph}), 132.6 (*C*-4a/8a^{Naph}), 129.8,

129.0, 127.8 (C^{Ar}), 127.6, 127.1 (C-5/8^{Naph}), 116.3 (CN), 86.3 (C-CN), 56.8 (α-CH^{Phe}), 52.5 (OMe), 51.5 (α-CH^{Leu}), 41.5 (β-CH$_2^{Leu}$), 40.0 (β-CH$_2^{Phe}$), 24.9 (γ-CH^{Leu}), 22.7 (δ-CH$_3^{Leu}$), 22.1 (δ-CH$_3^{Leu}$) ppm.

IR: ν=3291, 3066, 3032, 2957, 2871, 2213, 1742, 1686, 1600, 1576, 1531, 1333, 1291, 1229,1206, 1154, 725 cm^{-1}.

ESI-MS (*m*/*z*): 474.4 (76) $[M+H]^+$, 496.3 (100) $[M+Na]^+$.

ESI-HRMS (*m*/*z*) calculated for $[C_{27}H_{27}N_3NaO_5]^+$= 496.1843, found 496.1846.

$[\alpha]_D^{23}$= –48.1° (c=0.27, $CHCl_3$).

1.18. tert-Butyl-N-(3-chloro-1,4-dioxo-1,4-dihydronaphthalen-2-yl)-L-phenylalanyl-L-leucinate (7)

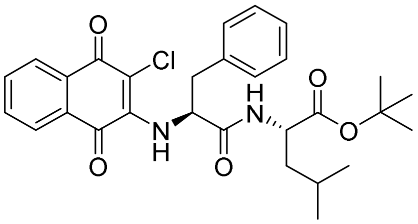

To the crude mixture of the deprotected dipeptide **A8** (20 mg, 60 μmol) were added triethylamine (13 μL, 90 μmol), 2,3-dichloro-1,4-naphthoquinone (20 mg, 90 μmol) and ethanol (1 mL). The mixture was stirred at 80 °C for 17 h. After cooling to room temperature, the solvent was removed. The residue was taken up in ethyl acetate (15 mL) and the organic phase was washed with water and brine (10 mL each). The organic phase was dried over sodium sulfate. Pure product (21 mg, 40 μmol, 67 %) was obtained as a red amorphous solid by chromatography (SiO_2, cyclohexane:ethyl acetate (9:1 to 1:9)).

R_f=0.17 (cyclohexane:ethyl acetate, 3:1).

^{1}H-NMR, COSY (300 MHz, $CDCl_3$) δ=8.11 (dd, *J*=7.6, 1.1 Hz, 1H), 8.01 (dd, *J*=7.8, 1.3 Hz, 1H, *H*-5/8^{Naph}), 7.71 (td, *J*=7.5, 1.5 Hz, 1H), 7.62 (td, *J*=7.6, 1.4 Hz, 1H, *H*-6/7^{Naph}), 7.32–7.17 (m, 5H, H^{Ar}), 6.41 (br d, *J*=7.5 Hz, 1H, NH^{Phe}), 6.14 (d, *J*=8.2 Hz, 1H, NH^{Leu}), 5.25 (dt, *J*=8.1, 6.5 Hz, 1H, α-CH^{Phe}), 4.47 (td, *J*=8.1, 5.5 Hz, 1H, α-CH^{Leu}), 3.21 (d, *J*=2.8 Hz, 1H, β-CH_2^{Phe}), 3.19 (d, *J*=2.8 Hz, 1H, β-CH_2^{Phe}), 1.62–1.42 (m, 3H, β-CH_2^{Leu}, γ-CH^{Leu}), 1.42 (s, 9H, ^{t}Bu), 0.91 (d, *J*=6.0 Hz, 3H, δ-CH_3^{Leu}), 0.90 (d, *J*=6.3 Hz, 3H, δ-CH_3^{Leu}) ppm.

^{13}C-NMR, HMBC, HSQC (75 MHz, $CDCl_3$) δ=180.2, 176.8 (C-1/4^{Naph}), 171.6 (C=O^{Leu}), 170.1 (C=O^{Phe}), 143.9 (C-3^{Naph}), 135.5 (C^{Ar}), 134.9, 132.9 (C-6/7^{Naph}), 132.3, 130.1 (C-4a/8a^{Naph}), 129.62, 129.0, 127.6 (C^{Ar}), 127.0, 126.9 (C-5/8^{Naph}), 82.3 (C^tBu) 58.9 (α-CH^{Phe}), 51.7 (α-CH^{Leu}), 42.7 (β-CH$_2^{Leu}$), 40.6 (β-CH$_2^{Phe}$), 25.0 (γ-CH^{Leu}), 22.8 (δ-CH$_3^{Leu}$), 22.3 (δ-CH$_3^{Leu}$) ppm.

IR: ν=3316, 2959, 2933, 2871, 1732, 1678, 1603, 1573, 1522, 1498, 1456, 1392, 1369, 1332, 1292, 1267, 1228, 1149, 722, 701 cm^{-1}.

ESI-MS (*m*/*z*): 469.4 (100) $[M - O^tBu+H_2O]^+$, 547.3 (40) $[M+Na]^+$.

ESI-HRMS (*m*/*z*) calculated for $[C_{29}H_{33}{}^{35}ClN_2NaO_5]^+$= 547.1971, found 547.1964; calculated for $[C_{29}H_{33}{}^{37}ClN_2NaO_5]^+$= 549.1941, found 547.1950.

$[\alpha]_D^{24}$= –18.3° (c=0.23, $CHCl_3$).

1.19. tert-Butyl-N-(3-cyano-1,4-dioxo-1,4-dihydronaphthalen-2-yl)-L-phenylalanyl-L-leucinate (8)

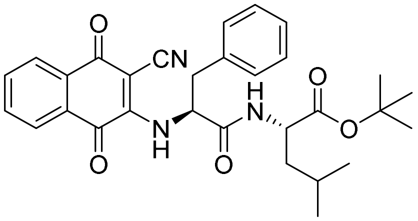

To a solution of dipeptide **A8** (8 mg, 24 µmol) and naphthoquinone **A9** (10.6 mg, 51 µmol) in ethanol (3 mL) was added triethylamine (6 µL, 45 µmol). The mixture was heated to 80 °C in a closed vessel for 18 h. After evaporation of the solvent, the residue was taken up in 50 mL of ethyl acetate and the organic phase was washed with water and 2 times brine (50 mL each). The organic phase was dried over sodium sulfate. Pure product (6 mg, 12 µmol, 50 %) was obtained by chromatography (SiO_2, petroleum ether:ethyl acetate (9:1 to 1:9)) as a yellow amorphous solid.

R_f=0.44 (cyclohexane:ethyl acetate, 2:1).

^{1}H-NMR, COSY (300 MHz, $CDCl_3$) δ=8.18 (dd, *J*=7.8, 1.3 Hz, 1H), 8.12 (dd, *J*=7.7, 1.3 Hz, 1H, *H*-5/8^{Naph}), 7.82 (m, 2H, *H*-6/7^{Naph}, N*H*^{Phe}), 7.71 (td, *J*=7.6, 1.4 Hz, 1H, *H*-6/7^{Naph}), 7.29–7.25 (m, 5H, *H*^{Ar}), 6.10 (d, *J*=7.9 Hz, 1H, N*H*^{Leu}), 5.33 (ddd, *J*=8.8, 7.3, 6.2 Hz, 1H, *α*-C*H*^{Phe}), 4.41 (td, *J*=8.0, 5.5 Hz, 1H, *α*-C*H*^{Leu}), 3.36 (dd, *J*=13.8, 6.2 Hz, 1H, β-C*H*$_2^{Phe}$), 3.21 (dd, *J*=13.8, 7.3 Hz, 1H, β-C*H*$_2^{Phe}$), 1.72–1.49 (m, 3H, β-C*H*$_2^{Leu}$, γ-C*H*^{Leu}), 0.92 (d, *J*=6.2 Hz, 3H, δ-C*H*$_3^{Leu}$), 0.91 (d, *J*=6.1 Hz, 3H, δ-C*H*$_3^{Leu}$) ppm.

^{13}C-NMR, HMBC, HSQC (75 MHz, $CDCl_3$) δ=179.2, 178.5 (*C*-1/4^{Naph}), 171.2 (*C*=O^{Leu}), 168.0 (*C*=O^{Phe}), 148.4, 136.1 (*C*-6/7^{Naph}), 134.5 (*C*$_q^{Ar}$), 133.5 (*C*-6/7^{Naph}), 132.7 (*C*^{Naph}), 129.8 (*C*^{Naph}), 129.7, 129.1, 127.9 (*C*^{Ar}), 127.7, 127.2 (C-5/8^{Naph}), 116.2 (CN), 86.5 (*C*-CN), 82.5 (*C*^{t}Bu), 56.8 (*α*-CH^{Phe}), 52.0 (*α*-CH^{Leu}), 41.9 (β-CH$_2^{Leu}$), 40.1 (β-CH$_2^{Phe}$), 28.1 (^{t}Bu), 25.0 (γ-CH^{Leu}), 22.7 (δ-CH$_3^{Leu}$), 22.3 (δ-CH$_3^{Leu}$) ppm.

IR: ν=3290, 2959, 2929, 2871, 2855, 2213, 1734, 1686, 1601, 1577, 1531, 1510, 1369, 1333, 1292, 1150 cm^{-1}.

ESI-MS (*m*/*z*): 538.4 (100) $[M+Na]^+$, 460.4 (77) [M - O^{t}Bu+H_2O]$^+$.

ESI-HRMS (*m*/*z*) calculated for $[C_{30}H_{34}N_3O_5]^+$=516.2493, found 516.2491.

$[\alpha]_D^{26}$= –12.4° (c=0.25, $CHCl_3$).

1.20. Benzyl-(1,4-dioxo-3-(phenethylthio)-1,4-dihydronaphthalen-2-yl)-L-phenylalanyl-L-leucinate (9)

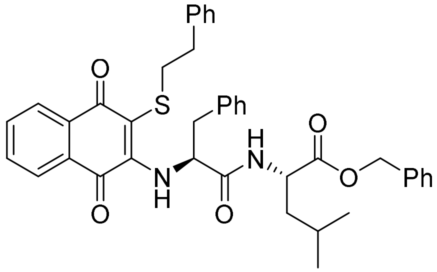

A solution of compound **1** (10 mg, 18 µmol), 2-phenylethanethiol (2.6 µL, 20 µmol) and triethylamine (2.7 µL, 20 µmol) in ethanol (2 mL) was heated to 70 °C for 12 h, another portion of 2-phenylethanethiol (2.6 µL, 20 µmol) and triethylamine (2.7 µL, 20 µmol) was added and heating was continued for 10 h. The solvent was removed in vacuo. Pure product (10 mg, 15 µmol, 83 %) was obtained as a red oil after chromatography (SiO_2, cyclohexane:ethyl acetate (4:1))

R_f=0.29 (cyclohexane:ethyl acetate, 3:1).

^{1}H-NMR, COSY (300 MHz, $CDCl_3$) δ=8.09 (dd, *J*=7.8, 1.2 Hz, 1H, *H*-5/8^{Naph}), 7.95 (dd, *J*=7.7, 1.3 Hz, 1H, *H*-5/8^{Naph}), 7.70 (td, *J*=7.5, 1.5 Hz, 1H, *H*-6/7^{Naph}), 7.60 (td, *J*=7.5, 1.4 Hz, 1H, *H*-6/7^{Naph}), 7.37–7.12 (m, 12H, *H*^{Ar}), 7.14–7.04 (m, 3H, *H*^{Ar}), 6.82 (d, *J*=7.8 Hz, 1H, N*H*^{Phe}), 6.03 (d, *J*=8.2 Hz, 1H, N*H*^{Leu}), 5.27 (q, *J*=7.0 Hz, 1H, α-C*H*^{Phe}), 5.08 (d, *J*=12.2 Hz, 1H, C*H*$_2$Ph), 5.07 (d, *J*=12.2 Hz, 1H, C*H*$_2$Ph), 4.69–4.59 (m, 1H, α-C*H*^{Leu}), 3.16–3.00 (m, 4H, SC*H*$_2$, β-C*H*$_2^{Phe}$), 2.86–2.78 (m, 2H, SCH$_2$C*H*$_2$), 1.65–1.39 (m, 3H, γ-C*H*^{Leu}, β-C*H*$_2^{Leu}$), 0.86 (d, *J*=6.2 Hz, 3H, δ-C*H*$_3^{Leu}$), 0.86 (d, *J*=6.1 Hz, 4H, δ-C*H*$_3^{Leu}$) ppm.

^{13}C-NMR, HMBC, HSQC (75 MHz, $CDCl_3$) δ=180.9 (*C*-1^{Naph}), 180.1(*C*-4^{Naph}), 172.3 (*C*=O^{Leu}), 170.7 (*C*=O^{Phe}), 140.1, 135.9, 135.3, 134.6, 133.4, 132.4, 130.9, 129.6, 129.0, 128.7, 128.7, 128.6, 128.5, 128.4, 127.5, 126.7, 126.6, 126.5, 112.2 (*C*SCH$_2$), 110.7, 67.2 (*C*H$_2$Ph), 59.8 (α-*C*H^{Phe}), 51.1 (α-*C*H^{Leu}), 41.7 (β-*C*H$_2^{Leu}$), 40.2 (β-CH$_2^{Phe}$), 36.4 (SCH$_2$*C*H$_2$), 35.6 (S*C*H$_2$CH$_2$), 24.9 (γ-*C*H^{Leu}), 22.8, 22.1 (δ-*C*H$_3^{Leu}$) ppm.

IR: ν=3310, 3064, 3029, 2957, 2870, 1741, 1673,1591, 1552, 1497, 1455, 1386, 1327, 1289, 1269 cm^{-1}.

ESI-MS (*m/z*): 661.5 (100) $[M+H]^+$, 683.3 (7) $[M+Na]^+$.

ESI-HRMS (*m/z*) calculated for $[C_{40}H_{41}N_2O_5S]^+$= 661.2731, found 661.2729.

$[\boldsymbol{a}]_D^{24}$= –80.0° (c=0.01, $CHCl_3$).

2. Table 1: Crystalllographic data and structure refinement for Benzyl-*N*-(3-chloro-1,4-dioxo-1,4-dihydronaphthalen-2-yl)-L-phenylalanyl-L-leucinate (1)

Empirical formula	$C_{32}H_{31}ClN_2O_5$
Formula weight	559.1
Temperature/K	193(2)
Crystal system	triclinic
Space group	P 1
a/ Å	4.8806(2)
b/ Å	12.4598(5)
c/ Å	22.9668(8)
α/°	84.104(3)
ß/°	84.261(3)
γ/°	87.680(3)
Volume/ $Å^3$	1381.64(9)
Z	2
ρ_{calc}/gcm^{-3}	1.344
μ/mm^{-1}	0.18
F(000)	588
Crystall size /mm^3	0.19 x 0.22 x 0.57
Radiation	Mo-K_α
2θ range for data collection /°	4 to 56
Index ranges	$-6 \leq h \leq 6$ $-16 \leq k \leq 14$ $-30 \leq l \leq 30$
Reflections collected	27655
Independent reflections	12755 (R_{int}= 0.0396)
Data/restraints/parameters	12755 / 714 / 803
Goodness-of-fit on F^2	1.040
Final R indexes [I>2σ(I)]	R1 = 0.0499, wR2 = 0.1315
Final R indexes [all data]	R1 = 0.0636, wR2 = 0.1483
Largest diff. peak/hole / $eÅ^{-3}$	0.59/-0.29

CCDC 1981158 contains the supplementary crystallographic data for this paper. These data are provided free of charge by the Cambridge Crystallographic Data Centre.

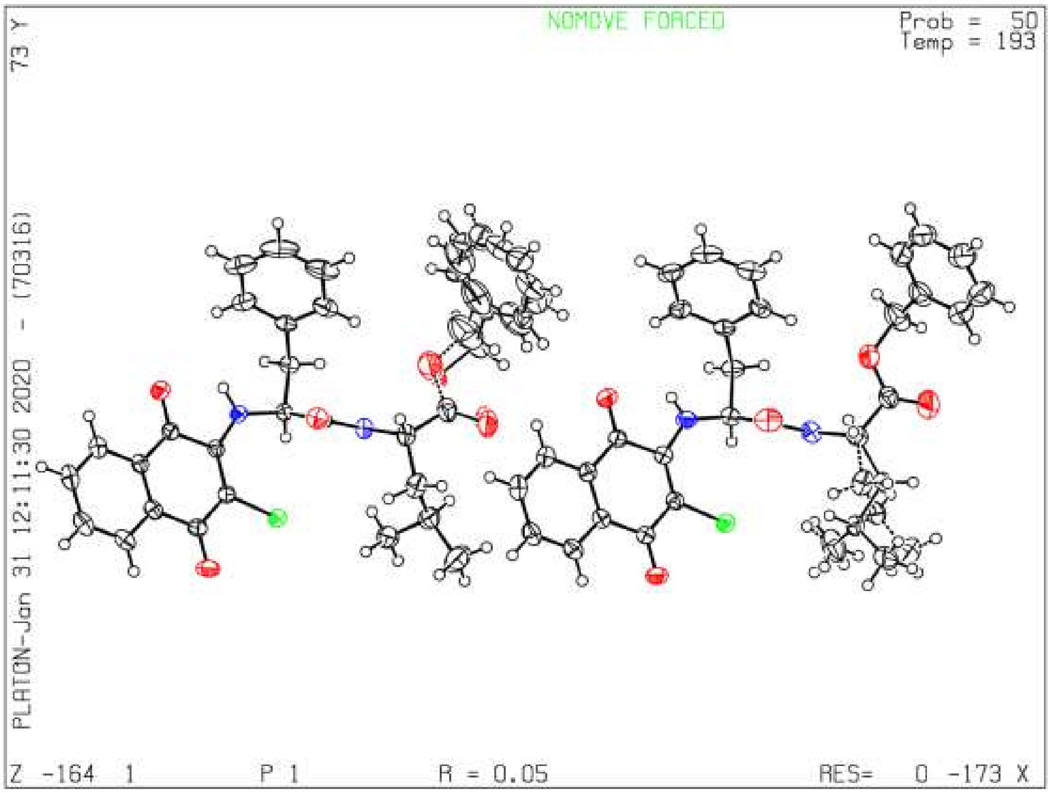
NOMOVE FORCED
Prob = 50
Temp = 193
73 Y
PLATON-Jan 31 12:11:30 2020 - (70316)
Z -164 1
P 1
R = 0.05
RES= 0 -173 X

3. Mass spectrometry

Lyophilized rhodesain was reconstituted at 4 mg/mL in 50 mM NaAcetate, pH 5.5, 200 mM NaCl, 5 mM EDTA. For mass spectrometric analysis, the protein was further diluted in the same buffer (to a final concentration of 850 nM) and reduced with DTT for 1 h at room temperature. After the addition of the drugs at a final concentration of 0.1mM, samples were analyzed by LC-MS using a nanoAcquity UPLC system (Waters Corporation) coupled to a nano-ESI-Q-TOF mass spectrometer (Synapt G2-S HDMS, Waters Corporation). Rhodesain without compound served as control. Protein-drug complexes were loaded onto a 200 µm x 5 cm PepSwift Monolithsic PS-DVB column from Dionex (Thermo Scientific) using direct injection mode. For LC separation, two mobile phases were used. Mobile phase A contained 0.1% FA and 3% DMSO in ultrapure water, whereas mobile phase B consisted of 0.1% FA and 3% DMSO in ACN. A gradient of 10-90% mobile phase B was run over 7 minutes at a flow rate of 2000 nL/min. Column temperature was set to 45°C. After separation, the column was rinsed with 90% of mobile phase B and re-equilibrated at initial conditions. All MS analyses were conducted in positive-mode ESI.

4. Enzyme assays and hydrolysis assays

Expression of rhodesain:

Rhodesain was expressed and purified from *Pichia pastoris* as described previously.[7,8]

Fluorometric enzyme assays:

The recombinantly expressed rhodesain (0.9 mg/mL) was diluted 1:400 in enzyme buffer (50 mM sodium acetate, pH 5.5, 5 mM EDTA, 200 mM NaCl and 2 mM DTT) and preincubated for 1 h at room temperature. Enzymatic reactions were carried out with 5 µL of the rhodesain stock solution in 180 µL assay buffer (50 mM sodium acetate, pH 5.5, 5 mM EDTA, 200 mM NaCl, 0.005% Brij). 10 µL of the inhibitors (final conc.: 100–0.001 µM) were added from DMSO stocks. Reactions were initiated by the addition of 5 µL Cbz-Phe-Arg-AMC in DMSO (final conc.: 10 µM). Enzymatic reactions were monitored for 30 min within a Tecan Spark microplate reader (λ_{ex}: 380 nm/λ_{em}: 460 nm).

Fluorometric assays of the DENV NS2B/NS3 protease were performed as described previously.[10] The assay was carried out with 5 µL of the DENV NS2B/NS3 protease (4 µM) in 180 µL assay buffer (50 mM Tris, pH 9.0, 20% glycerol and 1 mM CHAPS). 10 µL of the inhibitors (final conc.: 20 µM) were added from DMSO stocks. The reaction was initiated with 5 µL of Boc-Gly-Arg-Arg-AMC as a substrate (final conc.: 100 µM) and monitored with a Tecan Infinite M200 Pro plate reader (λ_{ex}: 380 nm/λ_{em}: 460 nm). Fluorometric assays for cathepsin B and cathepsin L (Calbiochem, Merck Millipore) were performed as described previously.[7,8] Cbz-Phe-Arg-AMC was used as substrate (final conc.: 100 µM for cathepsin B, 6.5 µM for cathepsin L) in assay buffer (50 mM Tris, pH 6.5, 5 mM EDTA, 200 mM NaCl, 0.005% Brij).

Dilution assay:

Recombinantly expressed rhodesain (0.9 mg/mL) or cathepsin L (Calbiochem, Merck Millipore) were diluted 1:100 in enzyme buffer (rhodesain: 50 mM sodium acetate, pH 5.5, 5 mM EDTA, 200 mM NaCl and 2 mM DTT; cathepsin L: 50 mM Tris, pH 6.5, 5 mM EDTA, 200 mM NaCl and 2 mM DTT) and preincubated for 1 h at room temperature. 54 µL of the respective enzyme solution was treated with 6 µL of cpd. **1–4** or K11777[10] in DMSO to yield a final inhibitor concentration of 5×IC_{50}. The enzyme-inhibitor reactions were incubated for 15 min at room temperature to ensure complete inhibition. 1.2 µL of each reaction mixture was diluted with 57.3 µL of the respective assay buffer (rhodesain: 50 mM sodium acetate, pH 5.5, 5 mM EDTA, 200 mM NaCl, 0.005% Brij; cathepsin L: 50 mM Tris, pH 6.5, 5 mM EDTA, 200 mM NaCl, 0.005% Brij) to give a final inhibitor concentration of 0.1×IC_{50}. Both the 5×IC_{50} and the 0.1×IC_{50} dilutions were initiated by the addition of 1.5 µL Cbz-Phe-Arg-AMC in DMSO (final conc.: 10 µM for rhodesain, 6.5 µM for cathepsin L) and monitored with a Tecan Infinite M200 Pro plate reader (λ_{ex}: 380 nm/λ_{em}: 460 nm).

Dialysis assay:

Dialysis experiments for rhodesain were performed using a custom-built dialysis chamber as described previously. [11] A 13 kDa MW cut-off dialysis tubing was used to separate a continuous flow from the reaction vessels of the instrument. 20 µL of rhodesain (2.3 µg/mL) was diluted with 740 µL enzyme buffer (50 mM sodium acetate, pH 5.5, 5 mM EDTA, 200 mM NaCl, 0.005% Brij) and 40 µL of cpd. **3**, **4** or K11777 in DMSO were added to the individual reaction. Activity control was performed by addition of pure DMSO. The final inhibitor concentration was chosen to be 20×IC_{50} in order to guarantee complete inhibition (cpd. **3**: 20 µM; cpd. **4**: 80 nM; K11777: 3 µM). After incubation for 30 min, the mixtures were transferred to the vessels of the dialysis chamber and dialyzed against a continuous flow of assay buffer containing 0.5% DMSO (400 mL/h). Samples (60 µL) were taken in

triplicates at different time points (0, 30, 60, 120 min) and reactions were initiated by the addition of 1.5 µL Cbz-Phe-Arg-AMC in DMSO (final conc.: 10 µM).

Hydrolysis assay – LC/MS:

Hydrolysis assays were performed as described previously.[12] To a solution of rhodesain (0.38 mg/mL) in 99 µL assay buffer (50 mM sodium acetate, pH 5.5, 5 mM EDTA, 200 mM NaCl and 2 mM DTT) 1 µL of either cpd. **1**, **2**, **6** or **8** in DMSO (20 mM) was added and the mixtures was incubated for 24 h at 25 °C. As negative reaction control, 99 µL assay buffer was treated with pure cpd. **1**, **2**, **6** or **8** in DMSO (20 mM) without rhodesain. The reactions were quenched by the addition of 100 µL acetonitrile and heat-inactivated for 5 min at 95 °C. Each solution was centrifuged for 10 min at 13 krpm and filtrated through a 0.22 µm syringe filter prior to LC/MS analysis. LC/MS analysis was performed using an Agilent 1100 series system with an Agilent Poroshell 120 EC-C18 column (150×2.10 mm, 4 µm; mobile phase: ACN/H_2O 70:30 +0.1% formic acid; flow rate: 0.7 mL/min).

Hydrolysis assay – UV-Vis:

To a solution of rhodesain (10 µg/mL) in 197.5 µL reaction buffer (50 mM sodium acetate, pH 5.5, 5 mM EDTA, 200 mM NaCl and 2 mM DTT, 5% DMSO) 2.5 µL of either cpd. **2**, **4**, **6** or **8** in DMSO (20 mM) was added and the absorbance was monitored for 2 h at 25 °C with a Tecan Spark 10M (λabs: 350-600 nm).

5. *T. b. brucei* cell survival assay

Toxicity of **2** against trypanosomes (*T. b. brucei* 449 cell line) were determined via an ATPlite assay as described previously[13–15] using the cellular ATP levels as a proxy for cell viability. **2** (5 mM stock solution in DMSO) was first diluted 1:3 in medium, followed by a 1:10 dilution step in a microplate and ten subsequent 1:2 dilution steps. 90 μL HMI-9 medium containing 2500 cells/mL were distributed in 96-well microplates (PerkinElmer). 10 μL of the 1:2 dilution step preparations of the tested compounds were added to the 90 μL cell suspension leading to final concentrations from 16.67 μM to 32.55 nM in the microplates. As a negative control, addition of 0.3% of DMSO corresponding to the highest DMSO concentration added by compound application was used. 10% DMSO was used as a positive control, since at this DMSO concentration, all cells die. Measurements were carried out as two sets of triplicates incubated at 37 °C for 24 h and 48 h. 50 μL of ATPlite 1 step solution (PerkinElmer) was added to each well of the microplate and luminescence measured at room temperature with an Infinite® M200 PRO plate reader (Tecan Trading AG). The measured values were plotted against the compound concentrations top yield the dose-response curve. The EC_{50} values were calculated using GraFit version 5.013 (Erithacus Software Ltd.).

6. NMR Spectra of the compounds

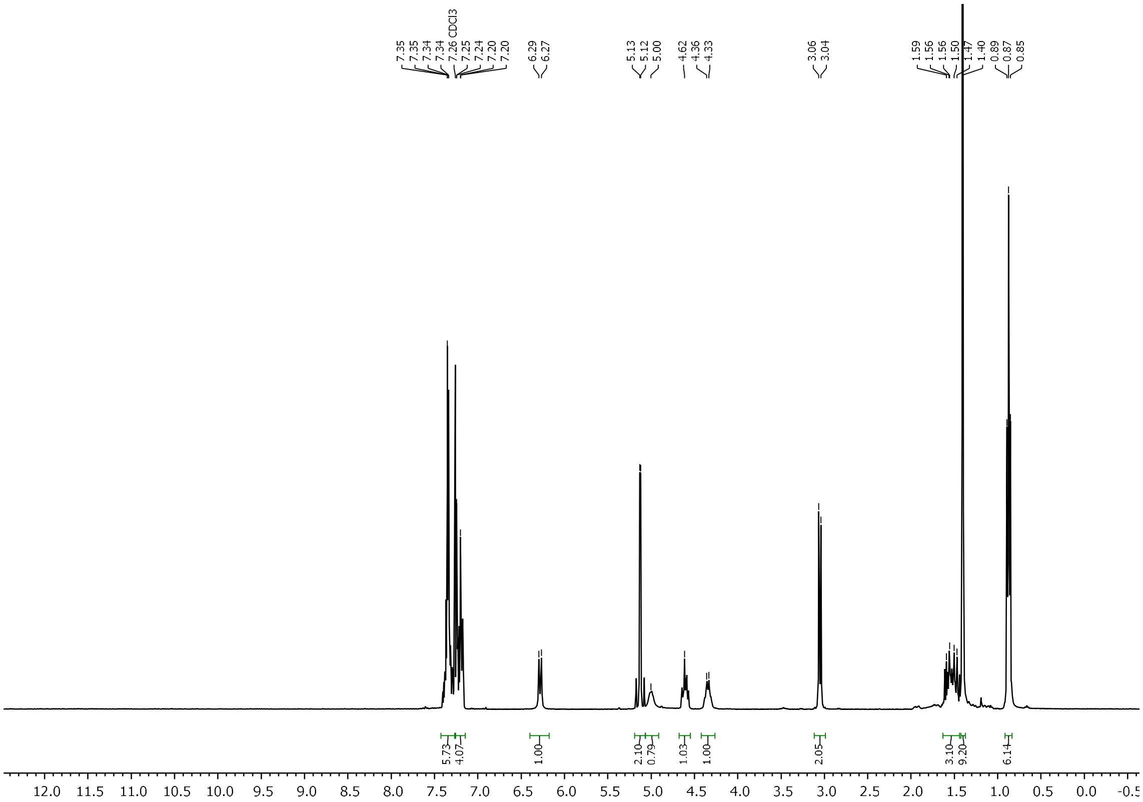

^{1}H-NMR (300 MHz, $CDCl_3$) spectrum of A1.

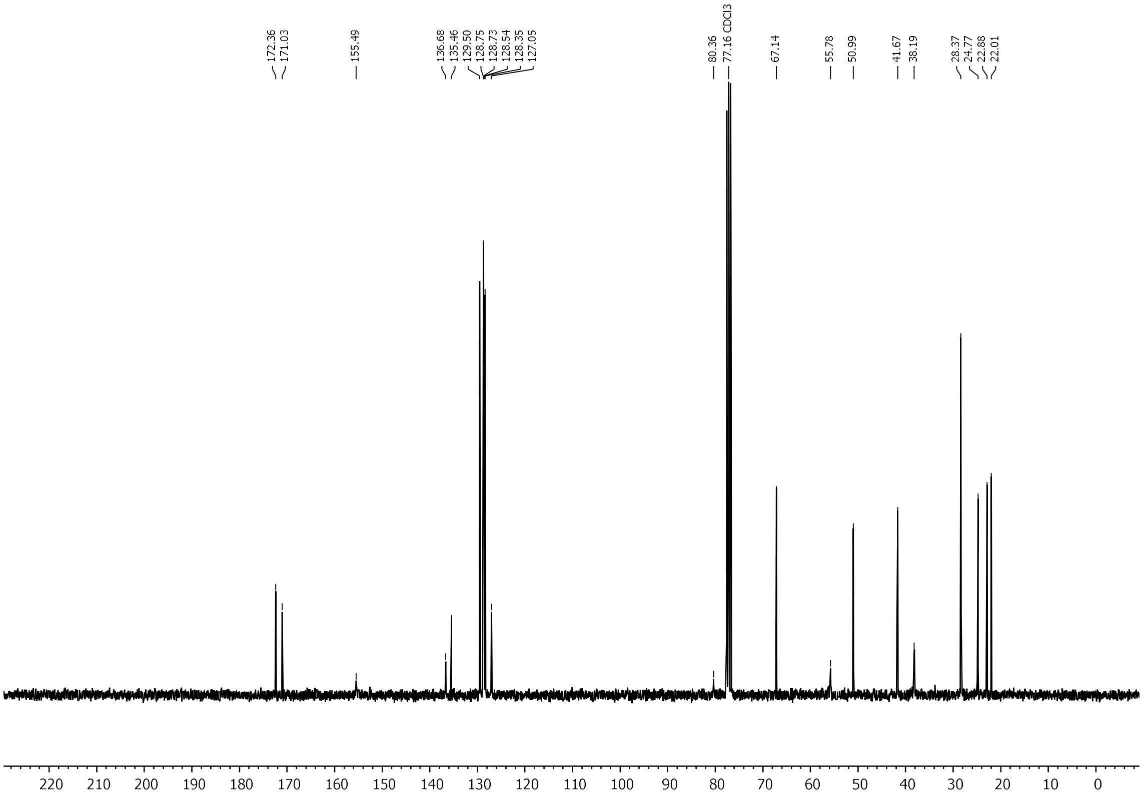

^{13}C-NMR (75 MHz, $CDCl_3$) spectrum of A1.

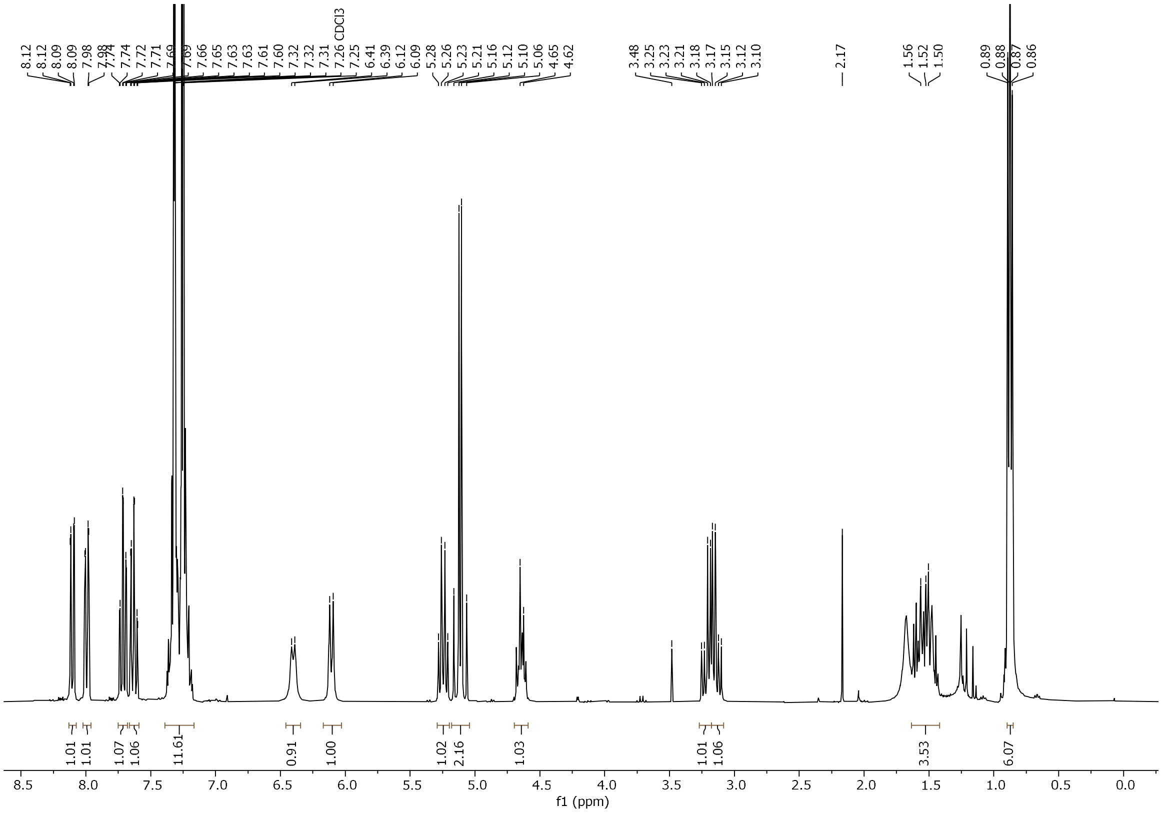

^{1}H-NMR (300 MHz, $CDCl_3$) spectrum of 1

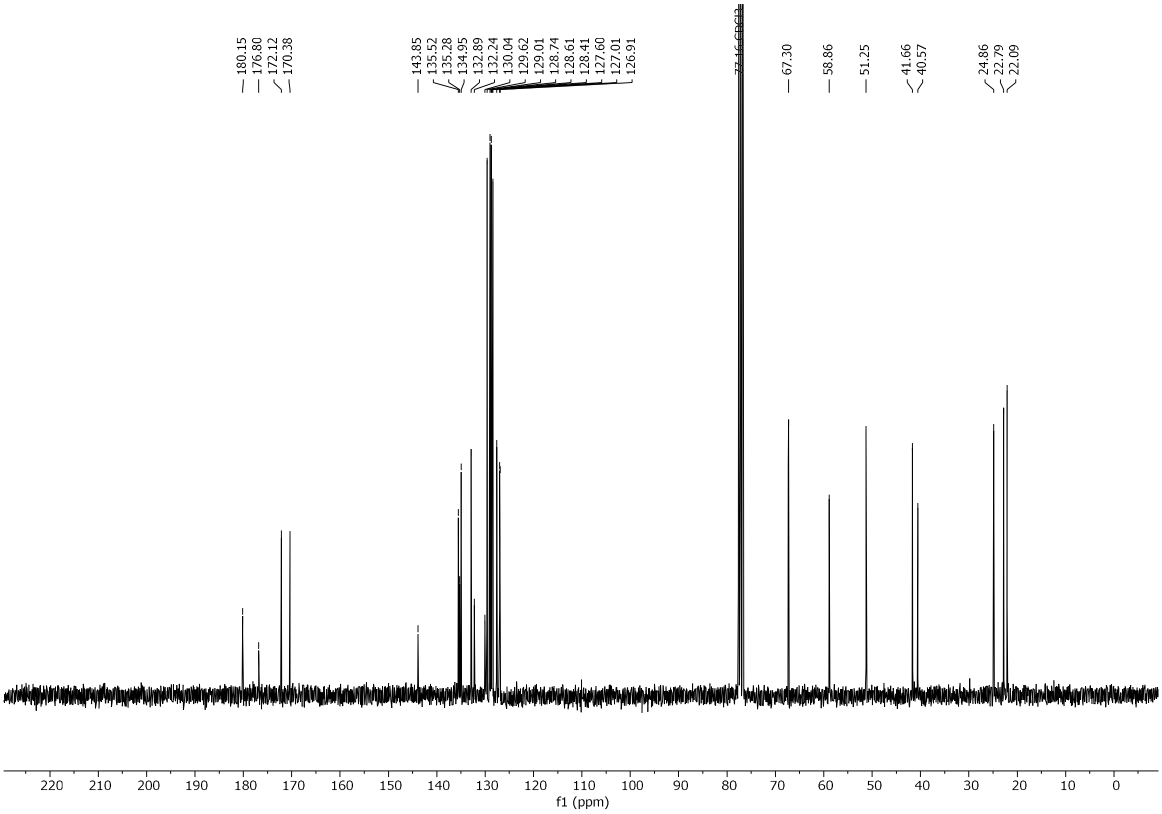

^{13}C-NMR (75 MHz, $CDCl_3$) spectrum of 1

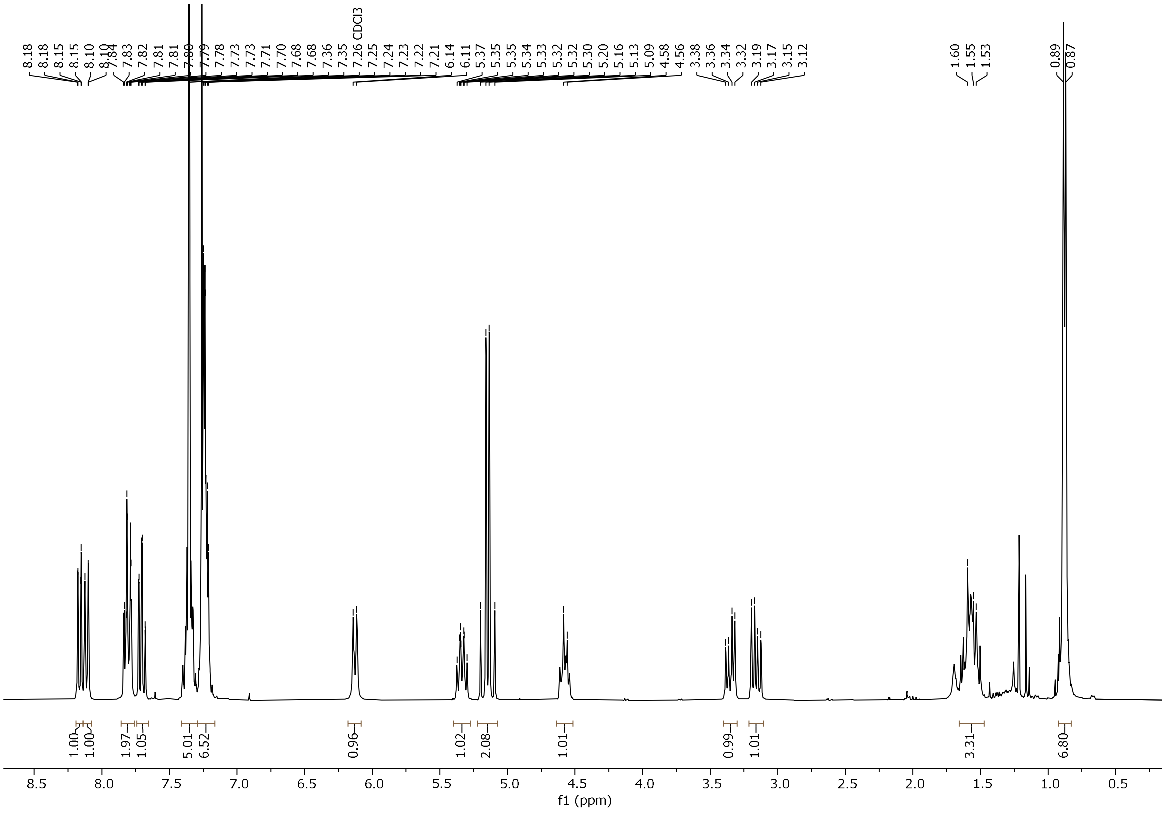

^{1}H-NMR (300 MHz, $CDCl_3$) spectrum of 2

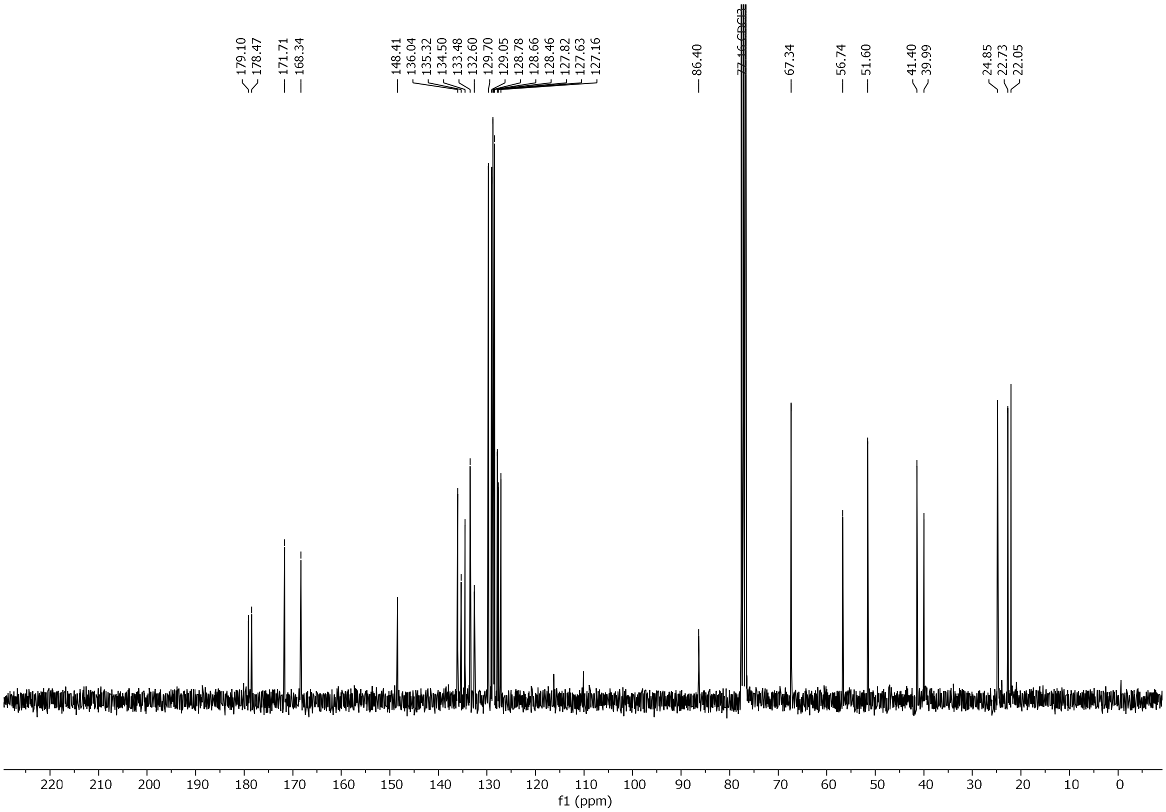

^{13}C-NMR (75 MHz, $CDCl_3$) spectrum of 2

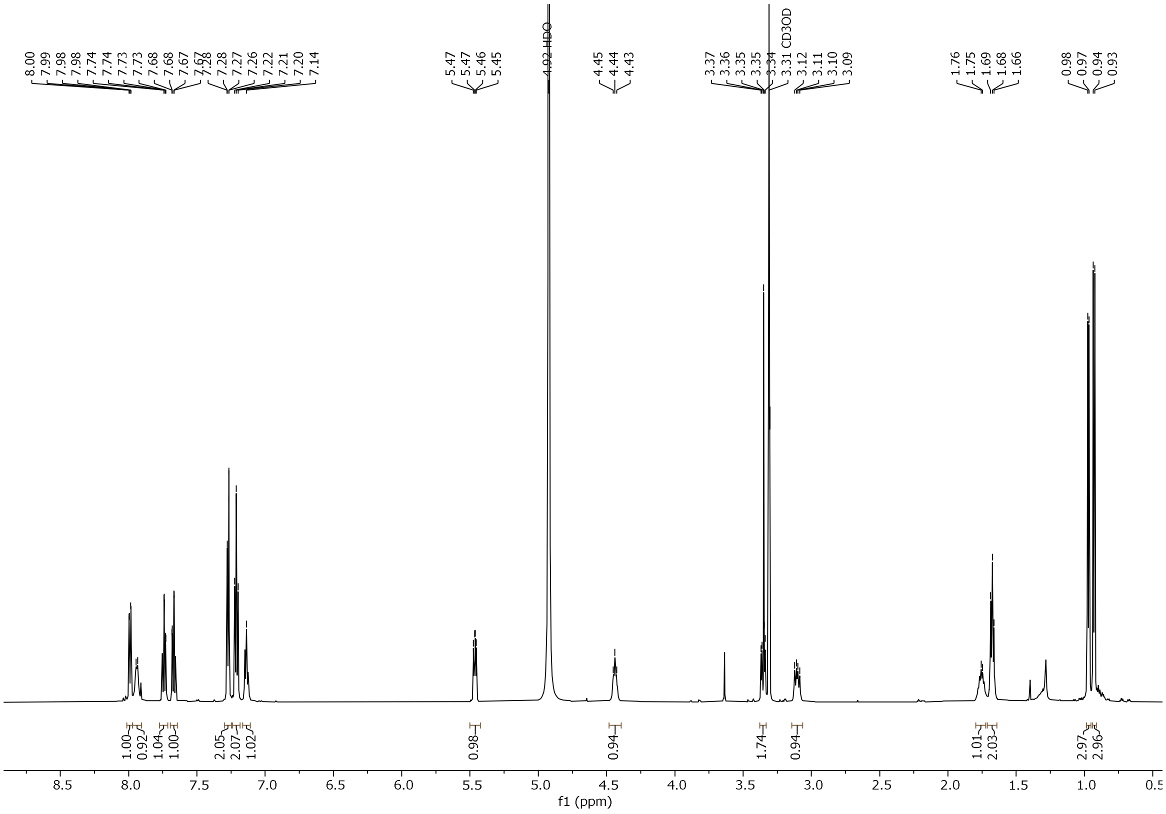

^{1}H-NMR (600 MHz, CD3OD) spectrum of 3

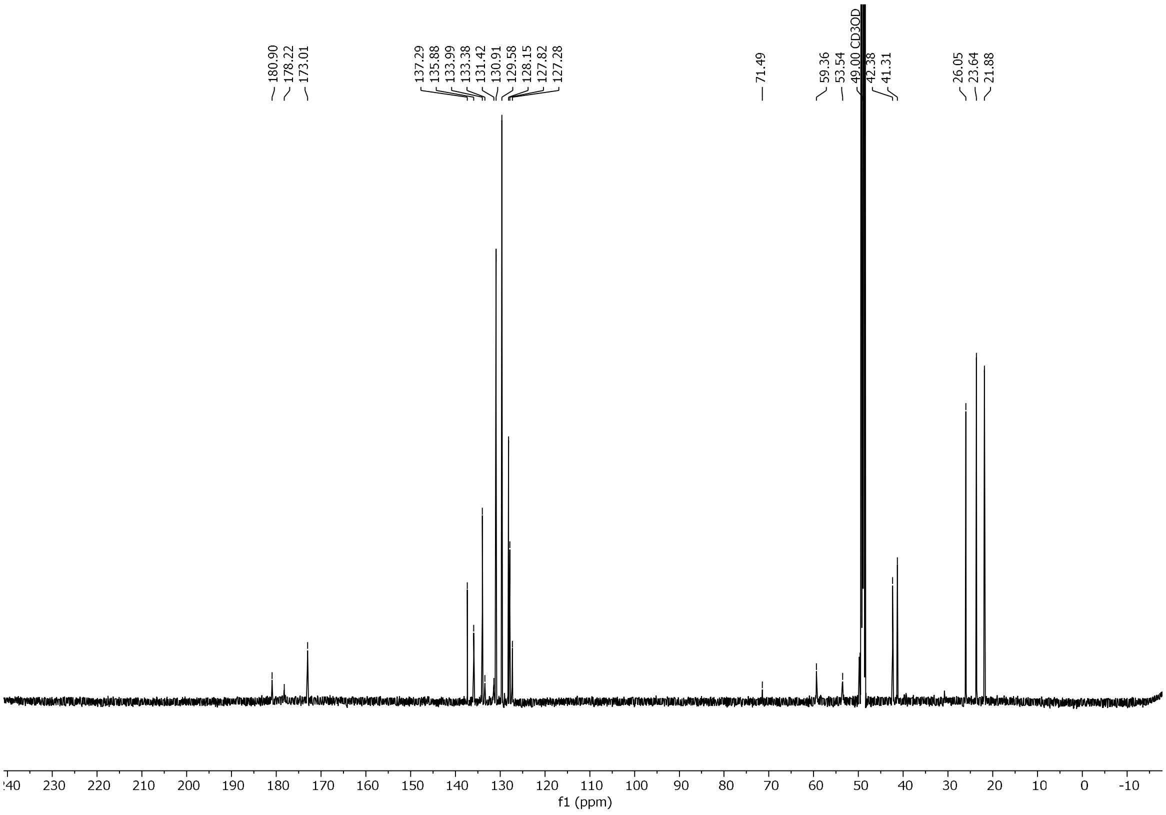

^{13}C-NMR (151 MHz, CD3OD) spectrum of 3

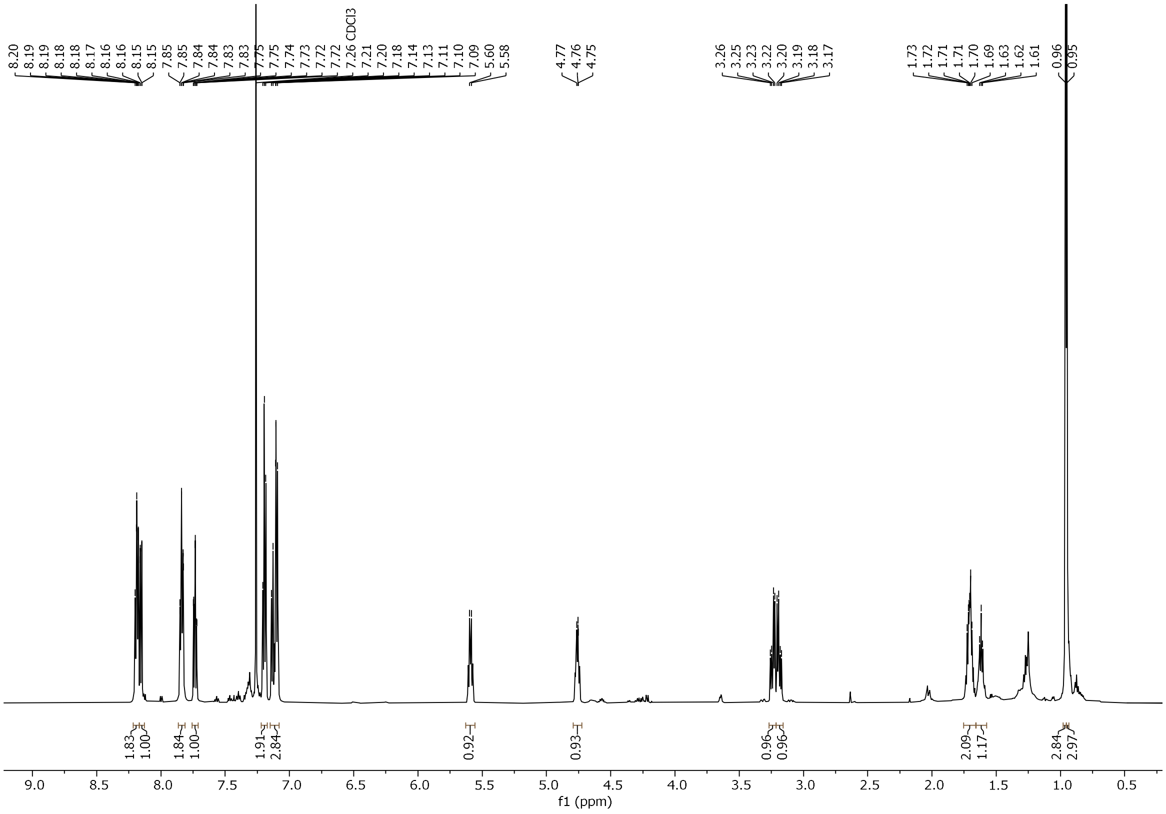

^{1}H-NMR (600 MHz, $CDCl_3$) spectrum of 4

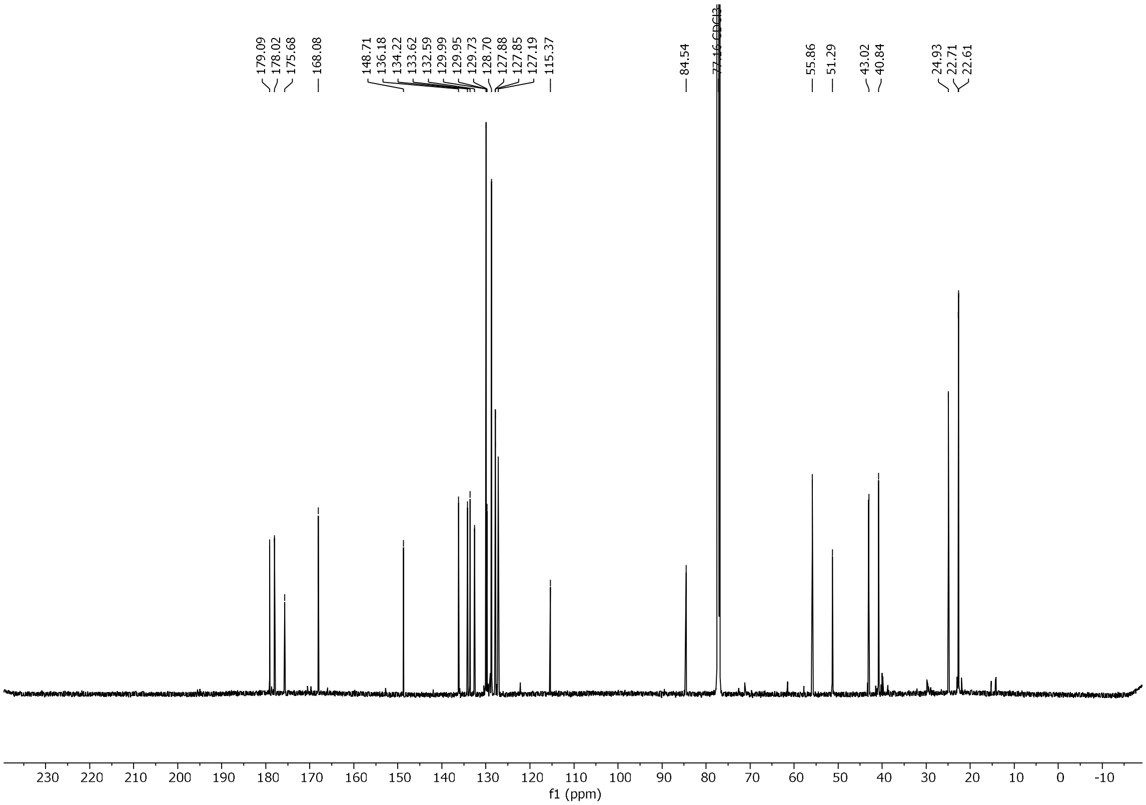

^{13}C-NMR (151 MHz, $CDCl_3$) spectrum of 4

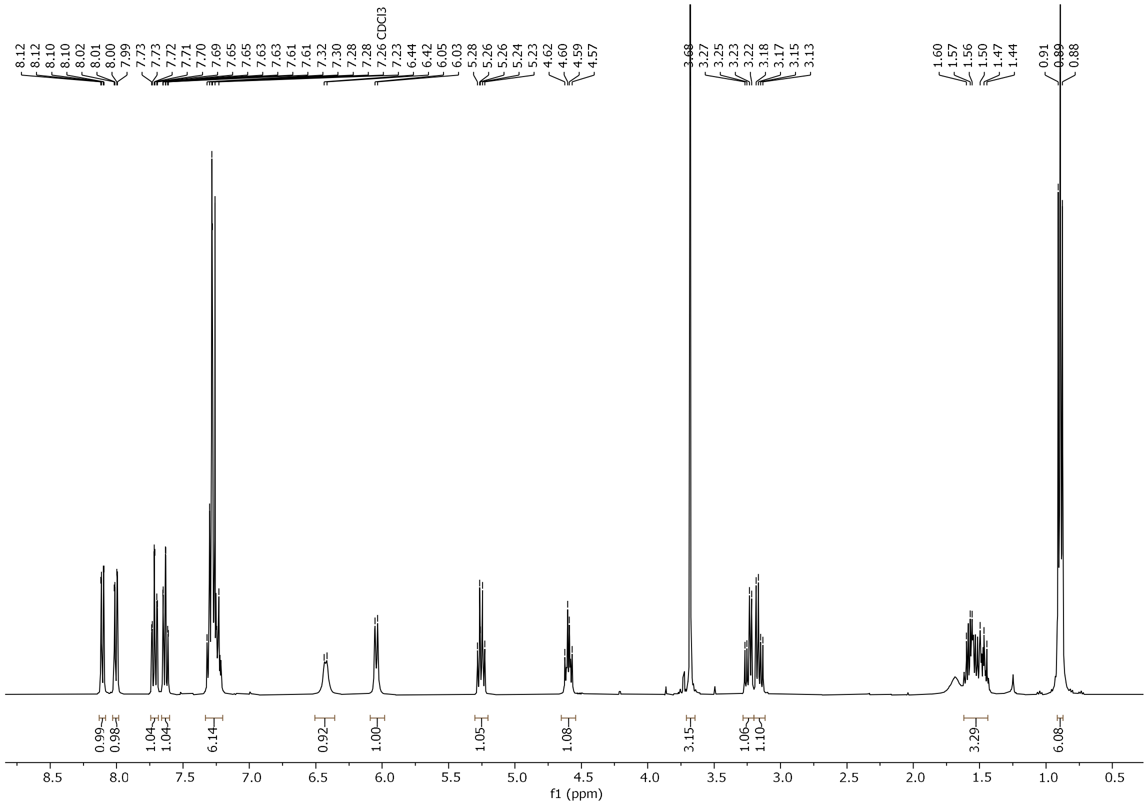

^{1}H-NMR (400 MHz, $CDCl_3$) spectrum of 5

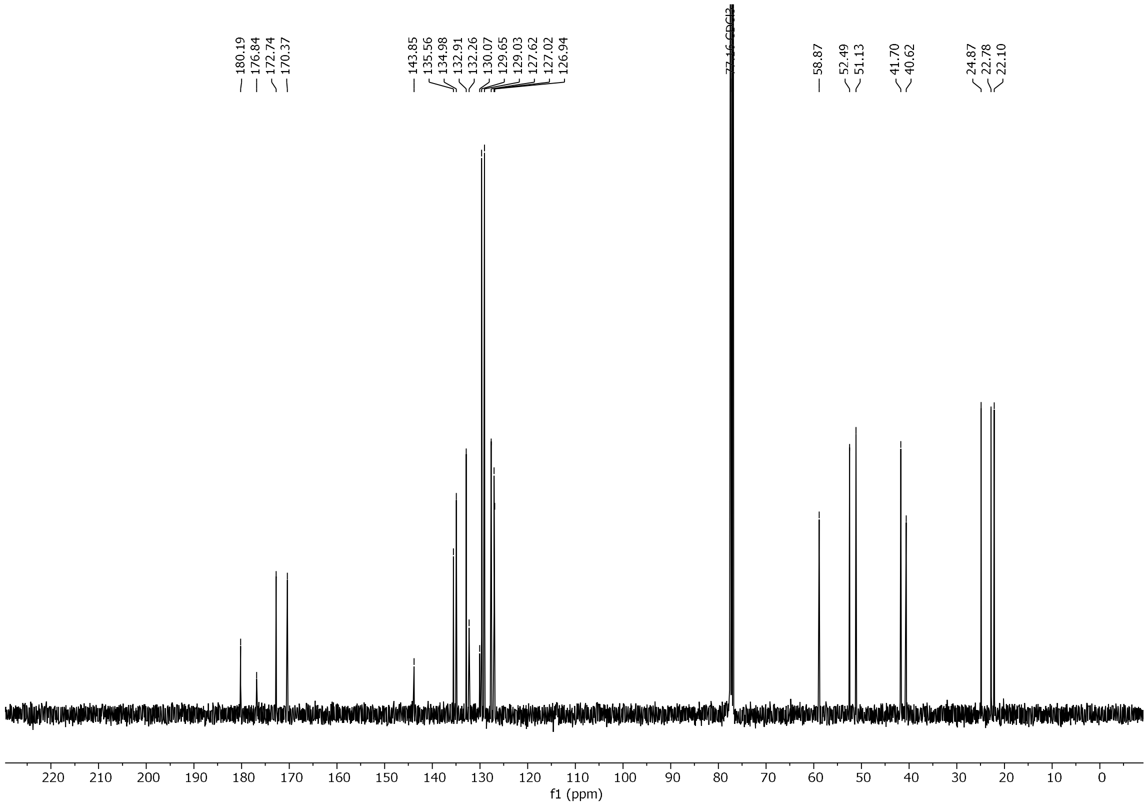

^{13}C-NMR (101 MHz, $CDCl_3$) spectrum of 5

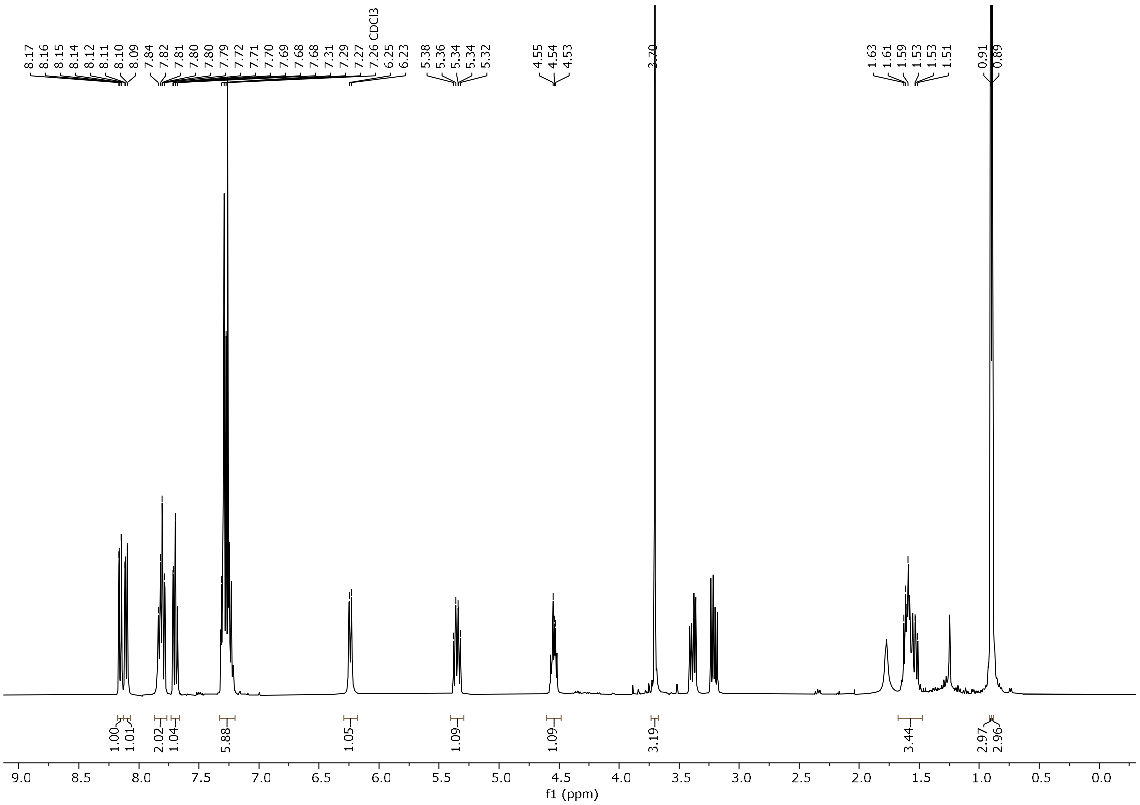

^{1}H-NMR (400 MHz, CDCl$_3$) spectrum of 6

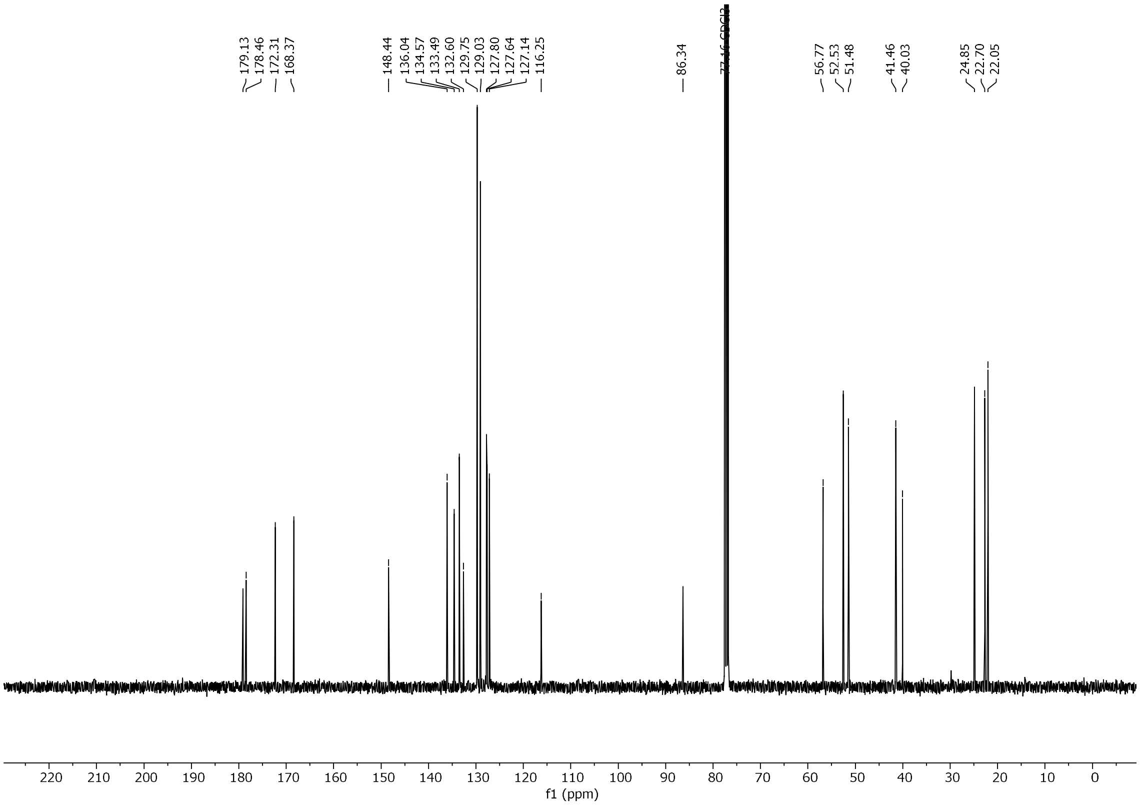

^{13}C-NMR (101 MHz, CDCl$_3$) spectrum of 6

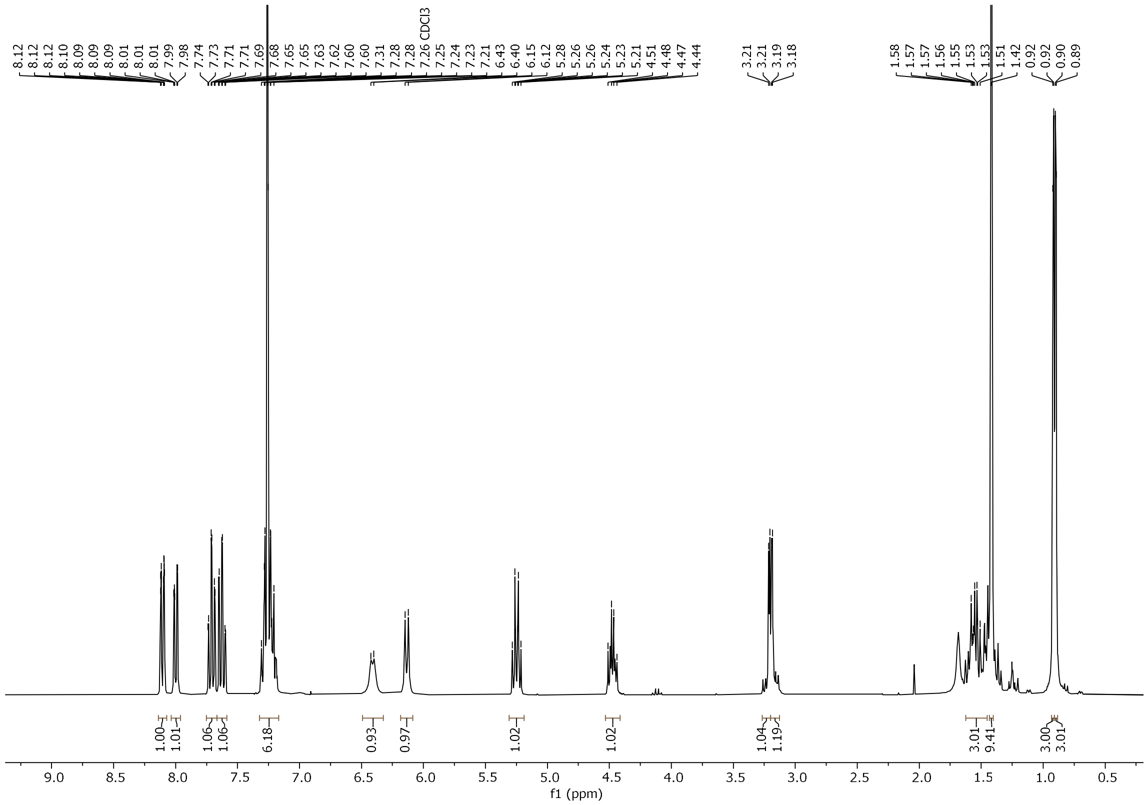

^{1}H-NMR (400 MHz, $CDCl_3$) spectrum of 7

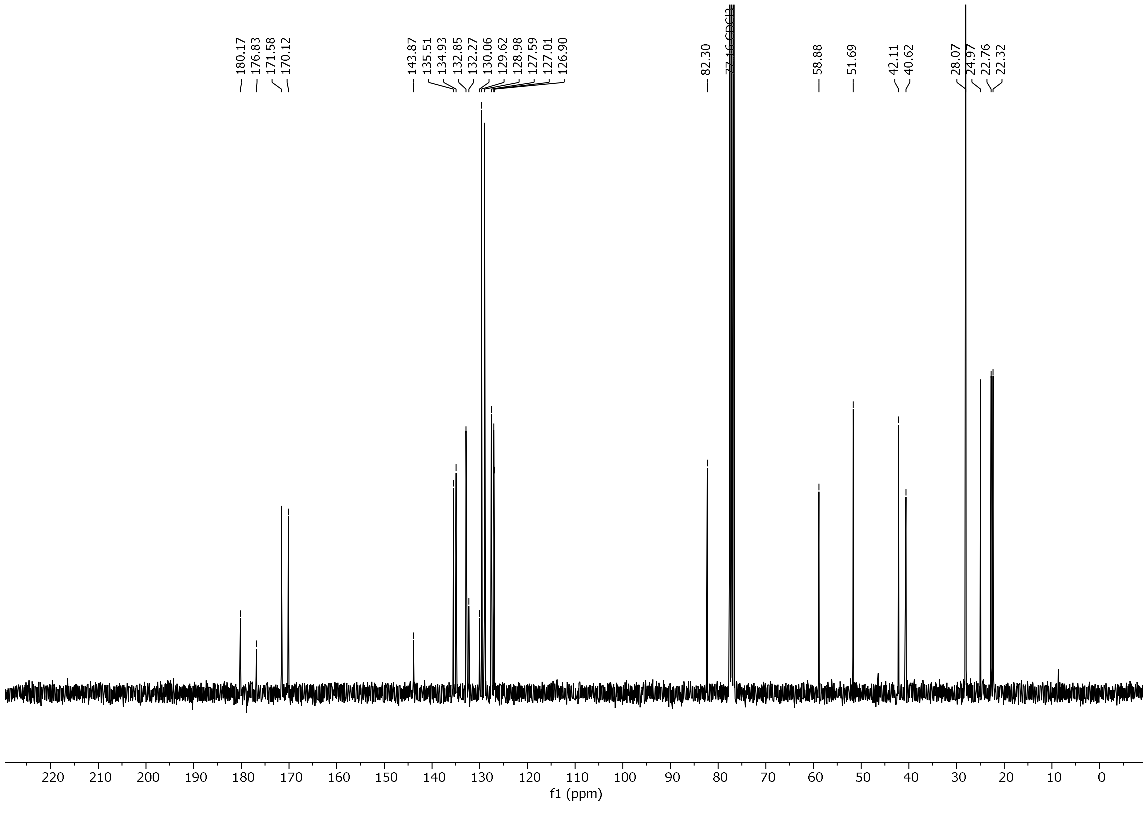

^{13}C-NMR (101 MHz, $CDCl_3$) spectrum of 7

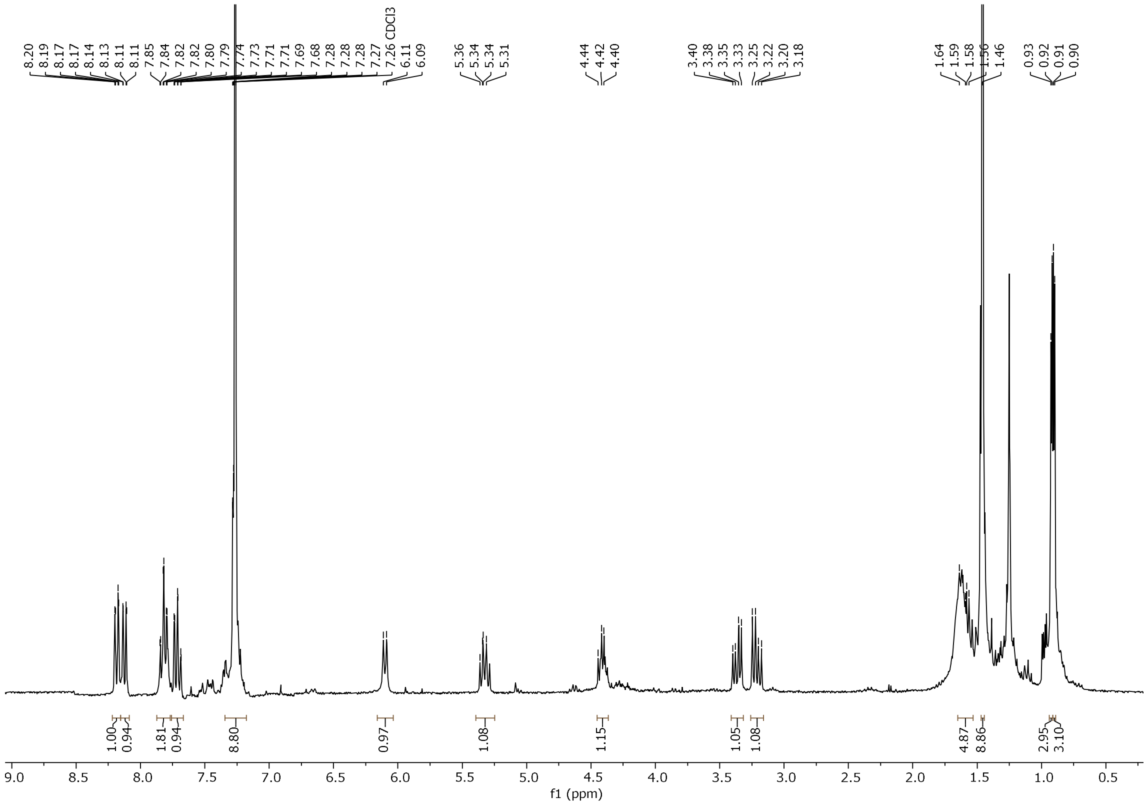

^{1}H-NMR (300 MHz, CDCl$_3$) spectrum of 8

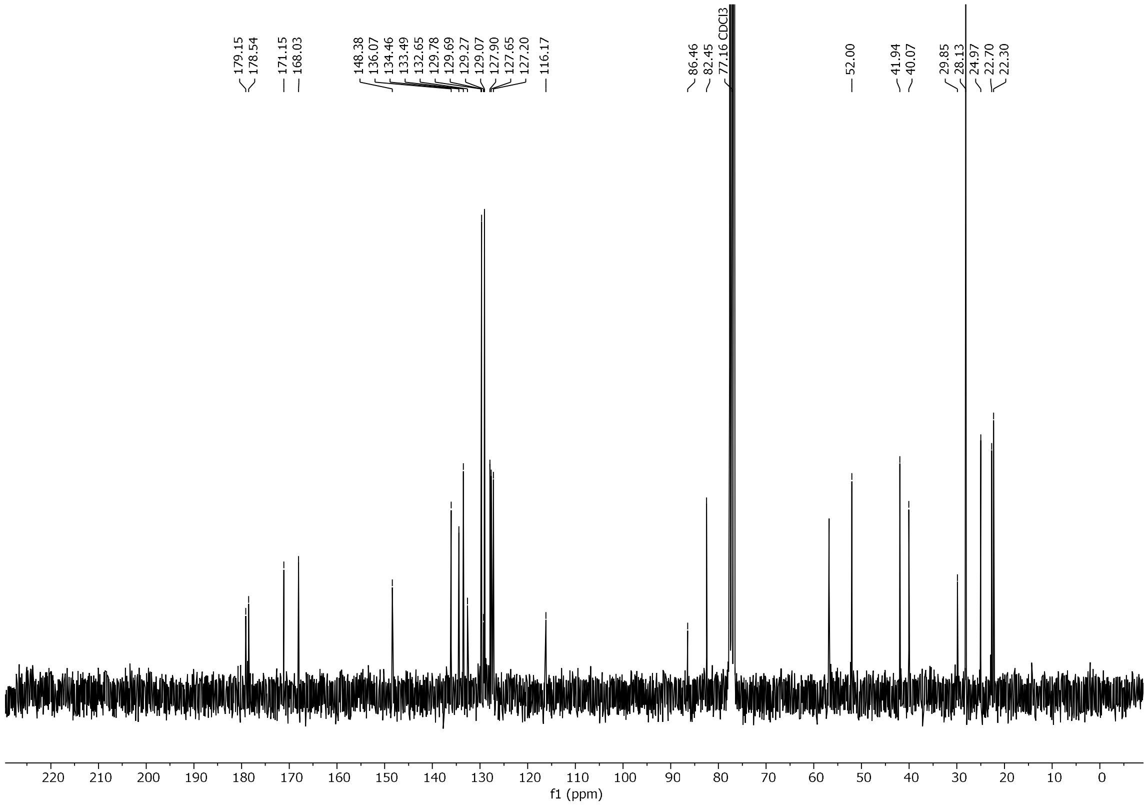

^{13}C-NMR (75 MHz, CDCl$_3$) spectrum of 8

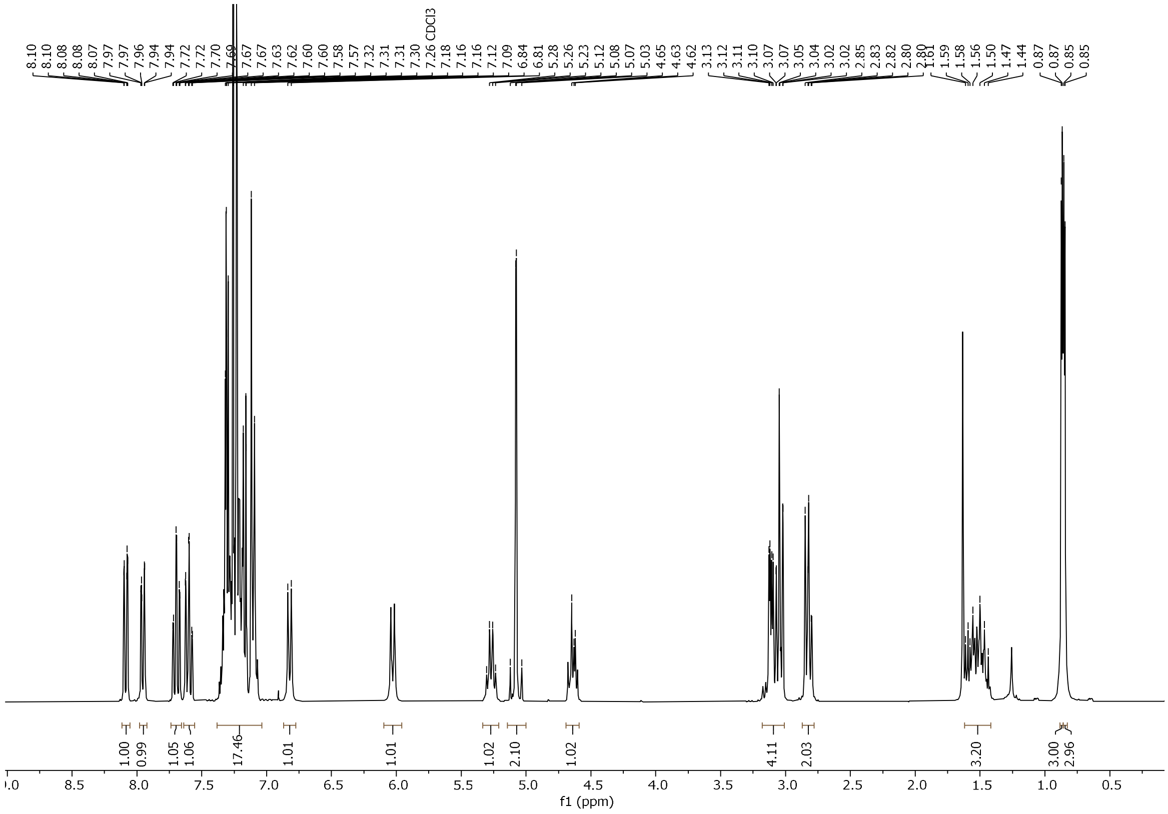

^{1}H-NMR (300 MHz, $CDCl_3$) spectrum of 9

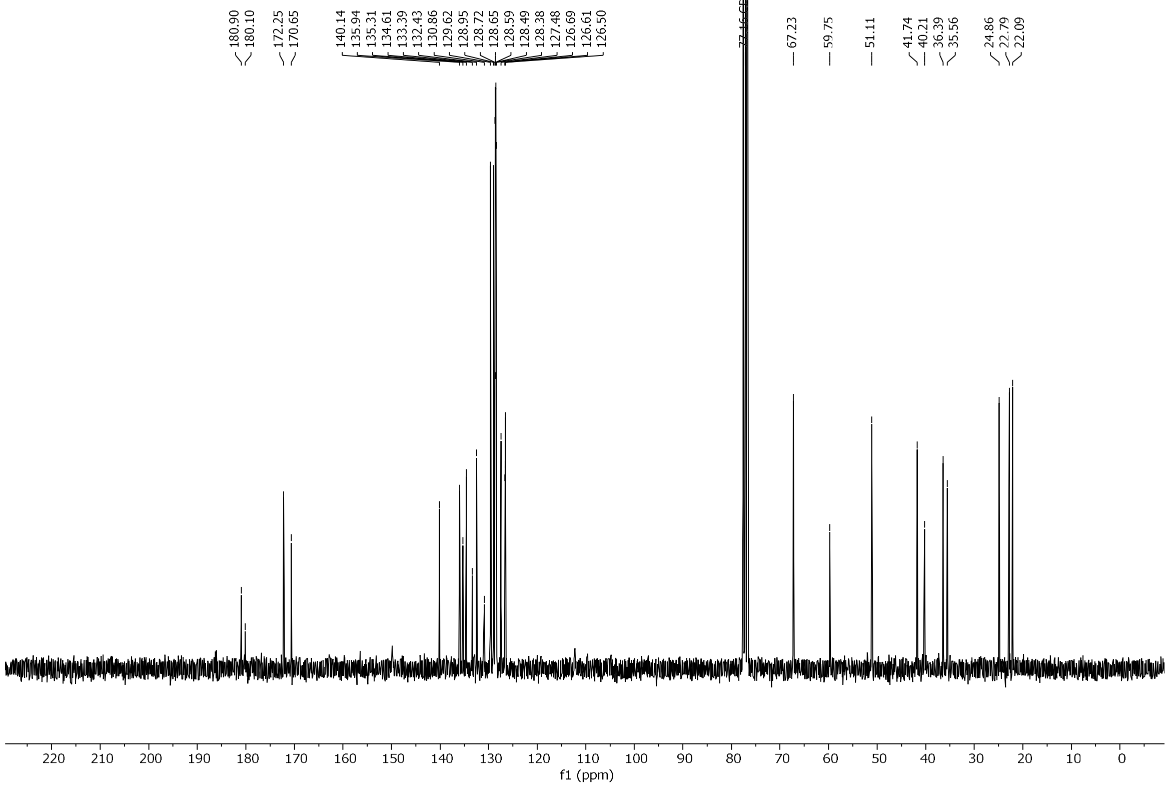

^{13}C-NMR (75 MHz, $CDCl_3$) spectrum of 9

7. References

1. Gottlieb, H.E.; Kotlyar, V.; Nudelman, A.J. NMR Chemical Shifts of Common Laboratory Solvents as Trace Impurities. *Org. Chem.* **1997**, *62*, 7512–7515.
2. Lippke, G.; Thaler, H. Die spezifische Drehung des Sorbits und des Sorbit-Molybdat-Komplexes. *Starch - Stärke* **1970**, *22*, 344–351.
3. Clausen, K.; Thorsen, M.; Lawesson, S.-O.; Spatola, A.F.J. Studies on amino acids and peptides. Part 6. Methods for introducing thioamide bonds into the peptide backbone: synthesis of the four monothio analogues of leucine enkephalin. *Chem. Soc. Perkin Trans. 1* **1984**, 785–798.
4. Han, G.; Tamaki, M.; Hruby, V.J.J. Fast, efficient and selective deprotection of the tert-butoxycarbonyl (Boc) group using HCl/dioxane (4 M). *Pept. Res.* **2001**, *58*, 338–341.
5. de Hatten, X.; Asil, D.; Friend, R.H.; Nitschke, J.R. Aqueous Self-Assembly of an Electroluminescent Double-Helical Metallopolymer. *J. Am. Chem. Soc.* **2012**, *134*, 19170–19178.
6. Neelgund, G.M.; Budni, M.L. Studies on Charge-Transfer Complexes of 2,3-Dicyano-1,4-naphthoquinone with Some Ring and *N*-Substituted Anilines. *Monatsh. Chem.* **2004**, *135*, 1395–1407.
7. Schirmeister, T.; Kesselring, J.; Jung, S.; Schneider, T.H.; Weickert, A.; Becker, J.; Wook, L.; Bamberger, D.; Wich, P.R.; Distler, U.; Tenzer, S.; Johe, P.; Hellmich, U.A.; Engels, B. Quantum Chemical-Based Protocol for the Rational Design of Covalent Inhibitors. *J. Am. Chem. Soc.* **2016**, *138*, 8332–8335.
8. Latorre, A.; Schirmeister, T.; Kesselring, J.; Jung, S.; Johé, P.; Hellmich, U.A.; Heilos, A.; Engels, B.; Krauth-Siegel, L.R.; Dirdjaja, N.; Bou-Iserte, L.; Rodríguez, S.; González, F.V. Dipeptidyl Nitroalkenes as Potent Reversible Inhibitors of Cysteine Proteases Rhodesain and Cruzain. *ACS Med. Chem. Lett.* **2016**, *7*, 1073–1076.
9. Millies, B.; Hammerstein, v.F.; Gellert, A.; Hammerschmidt, S.; Barthels, F.; Göppel, U.; Immerheiser, M.; Elgner, F.; Jung, N.; Basic, M.; Kersten, C.; Kiefer, W.; Bodem, J.; Hildt, E.; Windbergs, M.; Hellmich, U.A.; Schirmeister, T. Proline-Based Allosteric Inhibitors of Zika and Dengue Virus NS2B/NS3 Proteases. *J. Med. Chem.* **2019**, *62*, 11359–11382.
10. Kerr, I.D.; Lee, J.H.; Farady, C.J.; Marion, R.; Rickert, M.; Sajid, M.; Pandey, K.C.; Caffrey, C.R.; Legac, J.; Hansell, E.; McKerrow, J.H.; Craik, C.S.; Rosenthal, P.J.; Brinen, L.S. Vinyl Sulfones as Antiparasitic Agents and a Structural Basis for Drug Design. *J Biol Chem* **2009**, *284* (38), 25697–25703.
11. Ludewig, S.; Kossner, M.; Schiller, M.; Baumann, K.; Schirmeister, T. Enzyme kinetics and hit validation in fluorimetric protease assays. *Curr. Top. Med. Chem.* **2010**, *10*, 368–382.
12. Klein, P.; Johé, P.; Wagner, A.; Jung, S.; Kühlborn, J.; Tenzer, S.; Distler, U.; Anh Le, T.; Engels, B.; Hellmich, U.A.; Opatz, T.; Schirmeister, T. New cysteine protease inhibitors: S_NAr warheads and unexpected prodrug identification for the *Trypanosoma* protease rhodesain. *Molecules* **2020**, *25,* 1451*;* 1-22; *doi:10.3390/molecules25061451.*
13. Cunningham, M.P.; Vickerman, K. Antigenic Analysis in the *Trypanosoma brucei* Group, Using the Agglutination Reaction. *Trans. R. Soc. Trop. Med. Hyg.* **1962**, *56*, 48–59.
14. Crouch, S.P.; Kozlowski, R.; Slater, K.J.; Fletcher, J. The Use of ATP Bioluminescence as a Measure of Cell Proliferation and Cytotoxicity. *J. Immunol. Methods* **1993**, *160*, 81–88.
15. Wagner, A.; Le T.A.; Brennich, M.; Klein, P.; Bader, N. ; Diehl, E.; Paszek, D.; Weickhmann, A.K.; Dirdjaja, N.; Krauth-Siegel, R.L.; Engels, B.; Opatz, T.; Schindelin, H.A. Inhibitor-Induced Dimerization of an Essential Oxidoreductase from African Trypanosomes. *Angew. Chem. Int. Ed.* **2019**, *58*, 3640–3644.
16. Porte, V.; Thioloy, M.; Pigoux, T.; Métro, T.-X.; Martinez, J.; Lamaty, F. Peptide Mechanosynthesis by Direct Coupling of N-Protected α-Amino Acids with Amino Esters. *Eur. J. Org. Chem.* **2016**, *21* , 3505–3508.
17. Nuijens T.; Cusan C.; Kruijtzer J.A.W.; Rijkers D.T.S.; Liskamp, R.M.J.; Quaedflieg P.J.L.M. Versatile Selective α-Carboxylic Acid Esterification of N-Protected Amino Acids and Peptides by Alcalase. *Synthesis* **2009**, *5*, 809–814.
18. Filp, U.; Pekošak, A.; Poot, A.J.; Windhorst, A.D. Stereocontrolled [^{11}C]Alkylation of N-Terminal Glycine Schiff Bases To Obtain Dipeptides. *Eur. J. Org. Chem.* **2017**, *37*, 5592–5596.
